# NetrinG1^+^ Cancer-Associated Fibroblasts Generate Unique Extracellular Vesicles that Support the Survival of Pancreatic Cancer Cells Under Nutritional Stress

**DOI:** 10.1158/2767-9764.CRC-21-0147

**Published:** 2022-09-19

**Authors:** Kristopher S. Raghavan, Ralph Francescone, Janusz Franco-Barraza, Jaye C. Gardiner, Débora B. Vendramini-Costa, Tiffany Luong, Narges Pourmandi, Anthony Andren, Alison Kurimchak, Charline Ogier, Paul M. Campbell, James S. Duncan, Costas A. Lyssiotis, Lucia R. Languino, Edna Cukierman

**Affiliations:** 1 Doctoral program in Molecular Cell Biology and Genetics, Drexel University College of Medicine, Philadelphia, Pennsylvania.; 2 Cancer Signaling and Epigenetics Program, Institute for Cancer Research, Fox Chase Cancer Center, Philadelphia, Pennsylvania.; 3 Marvin and Concetta Greenberg Pancreatic Cancer Institute, Institute for Cancer Research, Fox Chase Cancer Center, Philadelphia, Pennsylvania.; 4 Department of Molecular & Integrative Physiology, University of Michigan Medical School, Ann Arbor, Michigan.; 5 Molecular Therapeutics Program, Institute for Cancer Research, Fox Chase Cancer Center, Philadelphia, Pennsylvania.; 6 Prostate Cancer Discovery and Development Program, Sidney Kimmel Cancer Center, Thomas Jefferson University, Philadelphia, Pennsylvania.

## Abstract

It is projected that in 5 years, pancreatic cancer will become the second deadliest cancer in the United States. A unique aspect of pancreatic ductal adenocarcinoma (PDAC) is its stroma; rich in cancer-associated fibroblasts (CAFs) and a dense CAF-generated extracellular matrix (ECM). These pathogenic stroma CAF/ECM units cause the collapse of local blood vessels rendering the tumor microenvironment nutrient-poor. PDAC cells are able to survive this state of nutrient stress via support from CAF-secreted material, which includes small extracellular vesicles (sEV). The tumor-supportive CAFs possess a distinct phenotypic profile, compared with normal-like fibroblasts, expressing NetrinG1 (NetG1) at the plasma membrane, and active Integrin α_5_β_1_ localized to the multivesicular bodies; traits indicative of poor patient survival. We herein report that NetG1^+^ CAFs secrete sEVs that stimulate Akt-mediated survival in nutrient-deprived PDAC cells, protecting them from undergoing apoptosis. Furthermore, we show that NetG1 expression in CAFs is required for the prosurvival properties of sEVs. In addition, we report that the above-mentioned CAF markers are secreted in distinct subpopulations of EVs; with NetG1 being enriched in exomeres, and Integrin α_5_β_1_ being enriched in exosomes. Finally, we found that NetG1 and Integrin α_5_β_1_ were detected in sEVs collected from plasma of patients with PDAC, while their levels were significantly lower in plasma-derived sEVs of sex/age-matched healthy donors. The discovery of these tumor-supporting CAF-EVs elucidates novel avenues in tumor–stroma interactions and pathogenic stroma detection.

**Significance:**

Results from this study identified two unique types of tumor-supporting CAF EVs, with evidence of these being detected in patients. Thus, this study facilitates a novel avenue to further dissect the subtleties of the tumor–stroma interactions responsible for PDAC homeostasis and progression, as well as the possibility of establishing future means to detect and monitor dynamic stroma staging.

## Introduction

Pancreatic ductal adenocarcinoma (PDAC) is the most common type of pancreatic cancer, accounting for approximately 90% of cases (1). PDAC has a sobering 11% overall 5-year survival rate (2, 3) and is currently the third most deadly cancer in the United States (4). Therefore, it is imperative to better understand PDAC biology to improve treatment options. In PDAC, a human tumor mass can, on average, be comprised of up to 70% stroma, which primarily includes cancer-associated fibroblasts (CAFs) and CAF-secreted interstitial extracellular matrix (ECM; ref. 5). Furthermore, the PDAC tumor microenvironment (TME) undergoes significant restructuring during tumor onset and progression (6, 7), which causes the collapse of local vessels and limits the access to hematogenous sources of nutrients. This stimulates a process of metabolic exchanges within the TME, resulting in the support of PDAC cell survival (8–11). It is therefore important to better understand how tumor-supportive CAF/ECM units provide local nutritional support to PDAC cells.

An important mode of paracrine communication between CAFs and PDAC cells is through the exchange of extracellular vesicles (EV; refs. 12, 13). EVs encompass a diverse range of secreted membranous transport structures that are generated by virtually all cells, are abundant in all bodily fluids, and serve an assorted array of functions (14). In the context of cancer, EVs are known to regulate various aspects of tumor development, including immune modulation, metabolic sustenance of cancer cells, and conditioning of the premetastatic niche (15–17). EVs accomplish these functions through intercellular transfer of their cargo, which include a variety of proteins, nucleic acids, and metabolites (17, 18). Furthermore, EVs contain transmembrane and surface glycoproteins that are integral for facilitating interactions with extracellular molecules (19, 20) as well as plasma membrane (PM)-exposed receptors (21).

This study focuses on heterogeneous populations of EVs in the 20–180 nm range, which can be grouped into two categories based on their mode of biogenesis. Small extracellular vesicle (sEV) populations are enriched with exosomes (80–180 nm) produced via endosomal trafficking to multivesicular bodies; and distinct nanoparticles (DNP) which are enriched in exomeres (20–80 nm) that are generated via “pinching off” from the plasma membrane (22, 23). Currently, dissecting the heterogeneous nature of EV populations constitutes a challenge. This is, in part, because the unique profile of each EV population is highly dependent on the type of cell, as well as on the functional state of the cell at the time EVs are generated. As such, the composition of an EV will reflect the functional state of its parent cell as well as its method of biogenesis, allowing EVs the potential to perform functions that modulate disease progression (24).

We recently identified NetrinG1 (NetG1), a glycosylphosphatidylinositol (GPI)-anchored cell surface protein most commonly observed in the central nervous system for its role in stabilizing glutamatergic synapses (25, 26), as a key driver of tumor-supportive CAF functions in PDAC (27). We reported that NetG1 ablation in CAF/ECM units modulates the CAF's metabolic secretory profile, and limits the nutritional support provided to PDAC cells (27). In addition, we previously published that there was a differential localization of the main fibronectin ECM receptor (detected in its active conformation), α_5_β_1_-integrin (Int.α_5_), in tumor-supportive pancreatic CAFs (e.g., Int.α_5_ trafficked intracellularly to the multivesicular body via the endosome) compared with tumor-restrictive pancreatic fibroblasts (e.g., Int.α_5_ enriched at the extracellular plasma membrane; ref. 28). Importantly, NetG1 expression and Int.α_5_ endosomal localization are maintained *in vitro* only if human PDAC CAFs are cultured as CAF/ECM functional units [i.e.*,* CAFs maintained within their cell-derived ECM (CDM)], which mimic the pathophysiologic conditions of the PDAC TME *in vivo* (27, 28).

In this study, we investigate the role of NetG1^+^ CAFs in generating tumor-supportive EVs. We identify a NetG1-dependent sEV function required to stimulate Akt-mediated survival of PDAC cells by preventing apoptosis induced by nutritional stress. In addition, we report that normal-like fibroblasts (NLF), which fail to express NetG1, generate EVs that are functionally reminiscent of EVs from NetG1-deficient CAFs. Moreover, we found that NetG1 expression in CAFs is responsible for generating sEVs capable of supporting the survival of nutrient-deprived PDAC cells. In addition, this study uncovers a novel NetG1^+^ EV subpopulation that is distinct from exosomes, and is enriched in the DNP population. To this end, proteomic and metabolic signatures were generated that confirm the successful enrichment of two types of unique EVs. Finally, we provide evidence to suggest that NetG1 and Int.α_5_ are enriched in EVs harvested from human PDAC patient plasma, yet scarce in plasma obtained from healthy age/sex-matched individuals.

Results from this study are significant in that they identify two unique types of EVs from tumor-supportive CAFs that can be detected in the blood of human patients with PDAC. Thus, this study further elucidates tumor–stroma interactions responsible for PDAC progression, as well as early evidence for establishing a means of using EVs to detect and monitor the PDAC TME.

## Materials and Methods

### Statement of Ethics

The authors state the work contained in this study was conducted in accordance with accepted ethical guidelines. All facilities within Fox Chase Cancer Center adhere to the International Society for Biological and Environmental Repositories and NCI Best Practices for Biospecimen Resources. All human samples used were donated following obtaining written and signed informed consent for the purpose of research and all were decoded for research usage. The studies were conducted in accordance with recognized ethical guidelines (e.g., Declaration of Helsinki, CIOMS, Belmont Report, U.S. Common Rule) and were approved by Fox Chase Cancer Center's Institutional Review Board.

### Key Resources Table

All key resources used in this study, including the sources and identifier codes are provided therein.

**Table:** 

Reagent	Source	Identifier
**Antibodies**		
Alix (1A12)	Santa Cruz Biotechnology	Catalog no. sc-53540, RRID:AB_673819
CD63 (MX-49.129.5)	SantaCruz Biotechnology	Catalog no. sc-5275, RRID:AB_627877
CD81 (B-11)	Santa Cruz Biotechnology	Catalog no. sc-166029, RRID:AB_2275892
Fibronectin antibody	Sigma-Aldrich	Catalog no. F0791, RRID:AB_476961
GAPDH	Abcam	Catalog no. ab9485, RRID:AB_307275
GFP (D5.1) XP	Cell Signaling Technology	Catalog no. 2956, RRID:AB_1196615
Hexokinase 1 (G-1)	Santa Cruz Biotechnology	sc-46695, RRID:AB_627721)
Histone 3	Cell Signaling Technology	Catalog no. 9715, RRID:AB_331563
Integrin alpha 5	Santa Cruz Biotechnology	sc-10729
NetrinG1-ligand (N1N3)	Genetex	Catalog no. GTX121508, RRID:AB_10721992
NetrinG1	Santa Cruz Biotechnology	Catalog no. sc-271774, RRID:AB_10707668
Phospho-Akt (Ser473)	Cell Signaling Technology	Catalog no. 4060, RRID:AB_2315049
Akt (pan)	Cell Signaling Technology	Catalog no. 2920, RRID:AB_1147620
PARP	Cell Signaling Technology	Catalog no. 9542, RRID:AB_2160739
SNAKA51 antibody	Gift from M Humphries	N/A
TSG101 [EPR7130(B]	Abcam	Catalog no. ab125011, RRID:AB_10974262
αSMA antibody	Sigma-Aldrich	Catalog no. A2547, RRID:AB_47670
Anti-Mouse IgG HRP	Cell Signaling Technology	Catalog no. 7076, RRID:AB_330924
Anti-Rabbit IgG HRP	Cell Signaling Technology	Catalog no. 7074, RRID:AB_2099233
Cy5 AffiniPure Donkey Anti-Rabbit IgG (H+L)	Jackson ImmunoResearch	Catalog no. 711-175-152, RRID:AB_2340607
Rhodamine Red-X (RRX) AffiniPure Donkey Anti-Mouse IgG (H+L)	Jackson ImmunoResearch	Catalog no. 715-295-151, RRID:AB_2340832
SYBR Green I	Thermo Fisher Scientific	Catalog no. S7563
**Chemicals**		
Protein Standard	Bio-Rad	1610374
4× Laemmli Sample Buffer	Bio-Rad	161-0747
Quick start Bradford 1× Dye Reagent	Bio-Rad	500-0205
Odyssey blocking buffer (PBS)	Li-COR Biosciences	927-70001
2-mercaptoethanol	Sigma-Aldrich	M3148-100ML
L-Ascorbic acid	Sigma-Aldrich	A92902-100G
Tween 20	Fischer Science	BP337-500
25% Glutaraldehyde solution in water	Sigma-Aldrich	G6257-1L
Paraformaldehyde 16% solution, EM grade	Electron Microscopy Sciences	15710
Uranyl acetate 2% solution	Electron Microscopy Sciences	22400-2
Ethanolamine	Sigma-Aldrich	E9508-1L
Dimethyl sulfoxide-for HPLC	Sigma-Aldrich	34869
ProLong Gold Antifade Mountant	Invitrogen	P36930
Sytox Blue	Invitrogen	S11348
Sytox Green	Invitrogen	S7020
Protease inhibitor	Thermo Fisher Scientific	36978
Phosphatase inhibitor	Thermo Fisher Scientific	A32957
**Cell culture**		
FBS	Peak Serum	PS-FB2
DMEM (High Glucose + Phenol Red)	Cell Culture Facility (FCCC)	N/A
DMEM (low Glucose+ Phenol Red)	Cell Culture Facility (FCCC)	N/A
DMEM (High glucose − Phenol Red)	Corning	17-205-CV
DMEM (low glucose − Phenol red)	Thermo Fisher Scientific	11054001
M3 media supplement	Incell	M300A
L-Glutamine	Corning	#25-005CI
Penicillin/Streptomycin	Corning	#30-002-CI
**Cell lines**		
Panc-1	ATCC	RRID: CVCL_0480
AsPC-1	ATCC	CRL-1682
Cancer-associated fibroblasts (patient derived)	Fox Chase Cancer Center	N/A
Tumor adjacent fibroblasts (patient derived)	Fox Chase Cancer Center	N/A
**Materials and machinery**		
Autoradiography film	Midwest Scientific	BX810
Film developer	AFP Imaging	Mini-Medical Series
Auto Fluorescent imager FluroChem E	Protein Simple	3184363
Confocal microscope	Nikon	Eclipse Ti2
Microscope Camera Orca-flash 4.0	Hamamatsu	C11440
0.22 μm PVDF syringe filter (33 mm)	Millipore	SLGV033RS
Polypropylene Ultracentrifuge tubes (11 × 34 mm)	Beckman Coulter	347357
Polypropylene Quick-Seal tubes (11 × 32 mm)	Beckman Coulter	344625
Amicon Ultra Centrifuge Filter Tubes	Millipore	UFC500324
Electron Microscopy Formvar coated Nickel (300 mesh) grid	Electron Microscopy Sciences	24928

### Parental Cell Lines

Patient-matched CAFs (and selected tumor adjacent fibroblasts) were collected from fresh surgical tissue and enzymatically digested and characterized using our well-established protocols (29, 30). PANC-1 and AsPC-1 were obtained from ATCC. hTERT-immortalized human pancreatic nestin-expressing (HPNE) cells and isogenic KRAS-HPNE (HPNE cells with E6/E7-KRASG12D mutations) cells (31), were also from ATCC. All experimental data include cells that were used within 3–15 passages after being thawed. Fibroblastic cells were authenticated for species of origin and reported genetic profiles (27, 28). All cells were tested regularly for *Mycoplasma* contamination using PCR detection.

### Engineered Cell Lines

Engineered cell lines used in this study were generated and validated as published in previous works include immortalized CAF and NLF [generated by knocking down the β_5_-integrin subunit (28)] cell lines, Ctl. and NetG1^KD^ CAF lines, mCherry-expressing PDAC cell lines, and NGL-1^KD^ PANC-1 cells (27–29). Cells engineered specifically for this project include parental CAFs (Ctl. and NetG1^KD^) overexpressing (OE) GFP. Briefly, eGFP was PCR amplified using Phusion HF polymerase reagent master mix, then cloned into Xbal/Xhol digested Fast AP dephosphorylated pLV-CMV-H4-puro vector. Ctl. and NetG1^KD^ CAFs were then transduced with pLV-CMV-H4-puro-GFP with 10 μg/mL polybrene. Cells were selected in media containing 2 μg/mL puromycin and GFP-expressing cells were further selected using flow cytometry. Parental NetG1^KD^ CAF cells OE ectopic NetG1 (NetG1^OE^) were generated as follows: NetG1 mRNA was isolated from CAFs and converted to cDNA using the SuperScript IV Reverse Transcriptase kit (Thermo Fisher Scientific). NetG1 cDNA was PCR amplified using primers that contained XbaI/XhoI overhangs, and the PCR product was cloned into the XbaI/XboI cut (New England Biolabs) and dephosphorylated (Fast AP, Thermo Fisher Scientific) pLV-CMV-H4-puro overexpression vector.

### Cell Culture and Condition Media Collection

All fibroblasts were cultured in DMEM containing 4.5 g/L glucose, 1.5 g/L NaHCO_3_, 10% FBS, 4 mmol/L l-glutamine (Corning), and 1% penicillin/streptomycin (Invitrogen). The methodology of our 7-day three-dimensional (3D)-cell derived ECM (also recognized as CDM) production has been published previously (30). At the completion of 3D matrix production, cells were rinsed twice with PBS and switched to a serum/EV-free DMEM (1% pen/strep, 4 μmol/L glutamine) and were sustained for 48 hours at 37°C, to generate conditioned media (CM). Upon completion of the conditioning period, CM was removed to be processed for further experiments, and the cells and CDMs were lysed for protein extraction (see methods below for depiction of metabolite extraction). All PDAC cell lines used in this study were maintained in 4:1 DMEM (1 g/L glucose, 110 mg/mL sodium pyruvate) M3 Base (INCELL) supplemented with 5% FBS, and 1% penicillin-streptomycin, until needed for experimental purposes.

### CAF/ECM Immunofluorescent Labeling and Image Acquisition

Fibroblast CDMs were imaged using immunofluorescent labeling as described previously (29). Cells and ECMs were immunolabeled using primary antibodies against fibronectin (rabbit IgG; 92.4 μg/mL) and α-smooth muscle actin (αSMA; mouse IgG; 75 μg/mL) for 1 hour at room temperature. Following primary immunolabeling, CDM cultures were washed with a solution of PBS and 0.05% TWEEN (PBST) three times for 5 minutes each. Biomarkers were labeled using host-specific secondary antibodies containing conjugated fluorophores for 1 hour at room temperature, so that fibronectin could be visualized at 647 nm, and αSMA at 568 nm. Following secondary antibody incubations, the CDMs were washed as before. SYBR Green (Invitrogen), a DNA stain that emits at 520 nm, was added to the cultures at (1:10,000) for 20 minutes at room temperature to visualize nuclei. Following SYBR Green treatment, CDM cultures were again rinsed and mounted onto a glass microscopy slides with Prolong Gold Anti-fade Mountant (Invitrogen). Samples were stored at 4°C for 48–96 hours to allow the mounting medium to set, and images were acquired with a Nikon Eclipse T*i*2 confocal microscope system at 60×. Excitation-emission spectra used for the following channels: FITC (488–520 nm), TRITC (540–580 nm), CY5 (640–670 nm). Six to eight images per coverslip were captured, covering a representative area of the entire coverslip. The top and bottom of each section being recorded was used to create a z-stack for each fluorescent channel. Images were then exported and processed via FIJI (32) for analysis as described in ref. 29.

### Media Fractionation, sEV Isolation, and Preparation of Functional CM

sEVs were purified from CM using differential centrifugation (33). Briefly, CM, collected as described above, was centrifuged for 10 minutes at 300 × *g*, followed by a 20-minute centrifugation at 2,000 × *g* to pellet cellular debris and dead cells. The resulting supernatant was spun for 30 minutes at 12,000 × *g* to pellet large vesicles and free-floating organelles. The new supernatant was filtered through a 0.22 μm polyvinylidene difluoride (PVDF) membrane using a syringe pump. The filtered sample was then either used as “functional conditioned media” for survival assays or transferred to a Beckman Optima TL-100 Ultracentrifuge (Beckman Coulter Life Sciences) for sEV isolation. To isolate sEVs, the filtered sample was spun down at 120,000 × *g* for 2 hours to pellet sEVs. The sEV supernatant was either used as an experimental condition or stored at 4°C to later isolate DNPs (see below). The pellet containing sEV was washed with 1 mL PBS and spun again at 120,000 × *g* for 2 hours to concentrate the pellet of sEVs. The resulting sEV pellet was resuspended in 50 μL PBS, and either used immediately for functional assays, or stored at −80°C for additional analytic characterization.

### Isolation of DNPs

DNP fractions were enriched from the supernatant of the sEV fraction (see above) by an additional ultracentrifugation stage that was adapted from Zhang and colleagues (34). The sample was spun at 120,000 × *g* for 16 hours to pellet DNPs (a subexosomal subset of extracellular vesicles). The DNP-containing pellet was washed with 1 mL PBS and spun again at 120,000 × *g* for 2 additional hours. The resulting DNP pellet was resuspended in 50 μL PBS, and either used immediately for functional assays, or stored at −80°C for additional analytic characterization.

### Human Plasma Acquisition and sEV Isolation

A total of 10 human plasma samples (6 patients with PDAC and 4 healthy donors) were used in this study. All individuals were white male of about 60 years of age. Samples were collected through Fox Chase Cancer Center's Institutional Biosample Repository Facility following a signed informed consent, using Health Insurance Portability and Accountability Act–approved protocols and exemption-approval of Fox Chase Cancer Center's Institutional Review board. Subjects’ identities were protected by classified coded identification numbers. Collected blood samples were processed to isolate plasma, and stored at −80°C. Once thawed, sEV isolation from plasma was performed similarly to that from cell-conditioned media with the following modifications: Plasma was diluted 1:1 with PBS, then centrifuged for 10 minutes at 500 × *g*, supernatant was collected and spun for 30 minutes at 2,000 × *g*. The resulting supernatant was spun for 45 minutes at 14,000 × *g*. This supernatant was filtered through a 0.22 μm PDVF membrane using a syringe pump. The filtered sample was spun down at 150,000 × *g* for 2 hours to pellet sEVs. The sEV pellet was washed with 1 mL PBS and spun again at 150,000 × *g* for 2 hours to concentrate the pellet of sEVs. The sEV pellet was resuspended in 150 μL PBS and stored at −80°C.

### Nanoparticle Quantification

Fractionated EV compositions were analyzed using two machines and their respective proprietary software: the ZetaView PMX-120 (ParticleMetrix), and the NanoSight NS300 (Malvern Pananalytical). Isolated EV samples were diluted 1:100 in sterile PBS (twice filtered through a 0.22 μm PDVF membrane) to a final volume of 1 mL. The diluted sample was then aspirated into a sterile 1 mL syringe and pumped into the Nanosight machine. Five 60-second videos were recorded wherein the sample was advanced progressively between each recording. These recorded videos were processed by the respective software, to produce a readout including the total particle count of the sample, in addition to batching the particle sizes with the concentration of particles for each size range.

### Cell Viability Assay

mCherry-expressing PDAC cells were rinsed 2× with PBS and switched to serum- and glutamine-free, low glucose (1.5 g/L) DMEM for 6 hours to subject cells to simulate pathophysiologic-like nutritional stress, prior to seeding in a 96-well clear-bottom black microplate (Grenier) at 7.5 × 10^3^ cells/well in rows B–G; cells were incubated at 37°C overnight. The following day, cell media was removed, and treatment conditions were administered to cells as described in figure legends. Separate conditions were arranged by column, providing six technical replicates with acellular wells at the top (A) and bottom (H) to act as media blanks per condition. Cells were incubated at 37°C for 48 hours posttreatment (unless stated otherwise) and gauged for cell viability. We defined “cell viability” as the ratio of live cells to dead cells. Live cells were measured via mCherry expression. Cell death was measured via treatment with Sytox Blue (Thermo Fisher Scientific). Sytox Blue was diluted in PBS and added to the media of all wells at a final concentration of 0.5 μmol/L and incubated in the dark at 37°C for 15 minutes, according to the manufacturer's protocol. After Sytox treatment, fluorescence was measured using a Tecan Spark 10M microplate reader (Tecan). Live cells (mCherry expression) were recorded using 540 nm (excitation)/590 nm (emission), and cell death (Sytox Blue) using 430 nm (excitation)/485 nm (emission); which was measured in that order to minimize residual autofluorescence between channels. Z-position and gain values were optimized to the positive control condition. Raw data for each fluorescence channel were processed by averaging the two blank values for each condition and subtracting the averaged blank value from the values of their respective conditions’ technical replicates. Blank-adjusted values for each channel were then used to divide the live cell value by the death cell value for each matched well. The cell viability ratios for each well were then normalized to the average of all control (PBS/untreated) values to identify each condition's fold change in cell viability compared to the control.

### PDAC/Fibroblast Direct Coculture

mCherry-expressing PDAC cells were switched to low glucose (1.5 g/L), serum/glutamine-free media for 24 hours and seeded at 7.5*10^3^ into a 96-well format containing fully confluent wells of preseeded fibroblasts and their secreted CDMs and incubated at 37°C for an additional 48 hours. After 48 hours, the plate was washed with PBS to remove dead cells and debris, before being analyzed on a Tecan Spark 10M microplate reader (Tecan). Numbers of PDAC cells were measured by quantifying mCherry expression attained using 540 nm (excitation)/590 nm (emission). PDAC levels were normalized to the average of all single-cultured PDAC cell values to identify each condition's fold change in PDAC cell count.

### Transmission Electron Microscopy

EVs were fixed in 2% paraformaldehyde (Electron Microscopy Sciences) and 20 μL of fixed EV suspension was carefully placed on top of a Formvar-coated Film, 300-mesh nickel grid (Electron Microscopy Sciences). To attain contrast stain, the grid was incubated onto a 30 μL droplet of PBS, twice, for 5 minutes, and on a 30 μL droplet of 2% uranyl acetate solution (Electron Microscopy Sciences) for 10 additional minutes at room temperature, Excess Uranyl acetate was removed and the gird was stored in the dark at room temperature for future use. For immune labeling, the grid was placed on a 30 μL droplet of Odyssey Blocking Buffer with a PBS-base (LI-COR) for 10 minutes at room temperature. The sample was then transferred onto a 30 μL droplet of primary antibody diluted in blocking buffer for 1 hour at room temperature. Antibodies used: CD81 (1 μg/mL), NetG1 (1 μg/mL), SNAKA51 (ref. 35; 0.225 μg/mL). Samples were rinsed three times with PBS for 5 minutes at room temperature prior to transferring onto a 30 μL droplet of corresponding secondary antibodies conjugated to either a unique sized gold particle or a unique quantum dot, diluted in blocking buffer, for 1 hour at room temperature. Samples were rinsed as before and incubated 2% uranyl acetate solution for 10 minutes as above. EVs were visualized under an JEOL 2100 transmission electron microscope (JEOL, Ltd.) at 60,000×–135,000× magnification.

### SDS-PAGE and Western Blot Analysis

SDS-PAGE and Western blot transfer were carried out as performed previously (27). Whole-cell lysates were prepared for Western Blotting by lysing 10^6^ cells in a 6-well format, using 100–200 μL of RIPA buffer as previously made (27). Protein concentrations of samples were determined using the Bradford assay (36) and working samples were normalized to an equal concentration (approximating 5 μg protein loaded per sample). To immunodetect protein, the following primary antibodies were used: Int.α_5_ (0.1 μg/mL), Alix (0.2 μg/mL), αSMA (7.5 μg/mL), CD63 (0.2 μg/mL), CD81 (0.067 μg/mL), GAPDH (0.05 μg/mL), GFP (0.014 μg/mL), Histone 3 (0.0006 μg/mL), phosho-Akt (0.03 μg/mL), pan-Akt (0.005 μg/mL), hexokinase 1 (0.8 μg/mL), NetrinG1 Ligand (1.0 μg/mL), NetG1 (1.6 μg/mL), PARP (0.39 μg/mL), TSG101 (0.44 μg/mL). Secondary antibodies used were as follows: anti-mouse HRP (0.03 μg/mL), anti-rabbit HRP (0.013 μg/mL). Immunoblots were visualized by treating membranes with Immobilon Western Chemiluminescent HRP substrate (Millipore), exposed, and developed on autoradiography film.

### sEV-mediated Transfer of GFP

sEVs were isolated from GFP-expressing CAFs, and then administered to PDAC cells to assess GFP transfer via Western blot analysis. AsPC-1 cells were rinsed 2× with PBS and switched to low glucose (1.5 g/L), serum/glutamine-free DMEM for 6 hours, before being plating at 10^3^ cells/well in a 6-well format. These cells were incubated for 24 hours in serum/glutamine-free media, before being rinsed 1× with PBS and treated with equal amounts of sEVs suspended in 2 mL PBS from GFP-expressing Ctl. or NetG1^KD^ CAFs. Equal volumes of sEVs from each condition were stored at −80°C to be used as input controls for Western blot analysis. Twenty-four hours posttreatment, supernatant was removed, cells were rinsed 2× and lysed with RIPA buffer and processed for Western blot analysis.

### Proteomic Sample Processing, Mass Spectrometry, and Spectra Analysis

Approximately 50 μg of EV proteins were reduced with 10 mmol/L dithiothreitol for 25 minutes, alkylated with 50 mmol/L indole-3-acetic acid for 30 minutes in the dark, and precipitated overnight with 80% acetone. The precipitated pellet was washed once with 80% acetone, resuspended in 6 M urea/2 M and digested using LysC and trypsin. Digested peptides were cleaned using stage tips, eluted with 70% acetonitrile and lyophilized. Samples were resuspended in 0.1% formic acid and separated with a Thermo Fisher Scientific RSLCnano Ultimate 3000 LC on a Thermo Fisher Scientific Easy-Spray C18 PepMap 75 μm × 50 cm C-18 2 μmol/L column. A 75-minute gradient of 2%–25% (30 minutes) acetonitrile with 0.1% formic acid was run at 300 nL/minute at 50°C. Eluted peptides were analyzed by a Thermo Fisher Scientific Q Exactive mass spectrometer utilizing a top 15 methodology in which the 15 most intense peptide precursor ions were subjected to fragmentation. The automatic gain control for MS1 was set to 3 × 10^6^ with a max injection time of 120 ms, the automatic gain control for MS2 ions was set to 1 × 105 with a max injection time of 150 ms, and the dynamic exclusion was set to 90 seconds.

### Proteomics Data Processing

Raw data analysis of label-free quantitation (LFQ) experiments was performed using MaxQuant software 1.6.1.0 (37) and searched using Andromeda 1.5.6.0 (38) against the Swiss-Prot human protein database (downloaded on April 24, 2019, 20402 entries). The search was set up for full tryptic peptides with a maximum of two missed cleavage sites. All settings were default and searched using acetylation of protein N-terminus and oxidized methionine as variable modifications. Carbamidomethylation of cysteine was set as fixed modification. The precursor mass tolerance threshold was set at 10 ppm and maximum fragment mass error was 0.02 Da. LFQ quantitation was performed with the following parameters: LFQ minimum ratio count: 1; global parameters for protein quantitation were as follows: label minimum ratio count: 1, peptides used for quantitation: unique, only use modified proteins selected and with normalized average ratio estimation selected. Match between runs was employed for LFQ quantitation and the significance threshold of the ion score was calculated on the basis of a FDR of <1%. MaxQuant normalized LFQ values were imported into Perseus software (1.6.2.3; ref. 39) and filtered in the following manner: kinases identified by site only were removed, reverse, or potential contaminants were removed. Protein LFQ values were log_2_ transformed and subjected to the Student *t* test comparing sEV and DNP fractions. Parameters for the Student *t* test were the following: S0 = 2, side both using Benjamini–Hochberg FDR <0.05. Data available in Supplementary Data S1—Proteomics.

### Protein Gene Ontology Analysis

Gene Ontology was performed using Metascape.org's open access gene annotation and analysis resources (40) following proteomic data processing. Statistically significant protein signatures identified using the Perseus software were entered into Metascape and run using their “express analysis” function. Using this list of proteins, Metascape's software identifies all statistically enriched terms including Gene Ontology/Kyoto Encyclopedia of Genes and Genomes terms, canonical pathways, and hallmark gene sets. Accumulative hypergeometric *P* values and enrichment factors were also calculated and used to filter results, which were clustered into a hierarchical tree based on Kappa-statistical similarities in gene membership. Then a 0.3 Kappa score was applied as the threshold to cast the tree into term clusters ranked by *P* value. Enrichment criteria including a minimum overlap value of 3, minimum enrichment value of 1.5, and a *P*-value cutoff of 0.01 were applied. A full report for each experiment is included in the spreadsheet file named Supplementary Data S1—Proteomics.

### sEV Boiling/Filtering

Crude metabolite contents of sEV and sEV supernatant fractions were collected by boiling samples. sEV and supernatant fractions were isolated as described above. Samples were resuspended in 500 μL sterile PBS and incubated at 100°C for 10 minutes. Samples were allowed to return to room temperature before being filtered through Amicon Ultra centrifuge filters, 3 kDa pore size (Millipore) for 30 minutes at 14,000 × *g*. Equal volumes of filtrate volume were collected, brought to a total volume of 900 μL sterile PBS and used in cell viability assays as described.

### Metabolite Sample Collection

CAF sEV and DNP fractions were isolated from conditioned media as described above. sEVs were resuspended in 200 μL PBS and 50 μL aliquots were taken for BCA protein quantification. The remaining volume was incubated with ice-cold methanol (80% by volume) for 10 minutes, inverting over dry ice. Sample was centrifuged at 17,000 × *g* for 10 minutes and supernatant was transferred to a clean tube. Metabolite supernatants were normalized to total protein content, and normalized samples were desiccated in a speed vacuum centrifuge and stored at −80°C until being processed. When ready, samples were suspended in a 50:50 mixture of methanol and water in HPLC vials for LC/MS-MS analysis and were run using the following protocol.

### Snapshot Metabolomics

Samples were run on an Agilent Technologies 1290 Infinity II LC-6470 Triple Quadrupole (QqQ) tandem mass spectrometer (MS/MS) system with the following parameters: 1290 Infinity II LC Flexible Pump (Quaternary Pump), 1290 Infinity II Multisampler, 1290 Infinity II Multicolumn Thermostat with 6 port valve and 6470 triple quad mass spectrometer. Agilent Masshunter Workstation Software LC/MS Data Acquisition for 6400 Series Triple Quadrupole MS with Version B.08.02 is used for compound optimization and sample data acquisition.

Solvent A is 97% water and 3% methanol 15 mmol/L acetic acid and 10 mmol/L tributylamine at pH of 5. Solvent C is 15 mmol/L acetic acid and 10 mmol/L tributylamine in methanol. Washing Solvent D is acetonitrile. LC system seal washing solvent 90% water and 10% isopropanol, needle wash solvent 75% methanol, 25% water. The materials used for the solvents were: GC-grade Tributylamine 99% (ACROS ORGANICS), LC/MS grade acetic acid Optima (Fisher Chemical), InfinityLab Deactivator additive, ESI-L Low concentration Tuning mix (Agilent Technologies), LC/MS grade solvents of water, and acetonritele, methanol (Millipore), isopropanol (Fisher Chemical).

An Agilent ZORBAX RRHD Extend-C18, 2.1 × 150 mm, 1.8 um and ZORBAX Extend Fast Guards for UHPLC are used in the separation. LC gradient profile is: at 0.25 mL/minute, 0–2.5 minutes, 100% A; 7.5 minutes, 80% A and 20% C; 13 minutes 55% A and 45% C; 20 minutes, 1% A and 99% C; 24 minutes, 1% A and 99% C; 24.05 minutes, 1% A and 99% D; 27 minutes, 1% A and 99% D; at 0.8 mL/minute, 27.5–31.35 minutes, 1% A and 99% D; at 0.6 mL/minute, 31.50 minute, 1% A and 99% D; at 0.4 mL/minute, 32.25–39.9 minute, 100% A; at 0.25 mL/minute, 40 minutes, 100% A. Column temperature is kept at 35°C, samples are at 4°C, injection volume is 2 μL.

6470 Triple Quad MS is calibrated with the ESI-L Low concentration Tuning mix. The source parameters are: Gas temp 150°C, Gas flow 10 L/minute, Nebulizer 45 psi, Sheath gas temp 325°C, Sheath gas flow 12 L/minute, Capillary −2,000 V, Delta EMV −200 V. Dynamic MRM scan type is used with 0.07-minute peak width and the acquisition time is 24 minutes. dMRM transitions and other parameters for each compound are listed in a separate sheet. Delta retention time of plus and minus 1 minute, fragmentor of 40 eV and cell accelerator of 5 eV are incorporated in the method.

### Statistical Analysis

Prism 7.05 (Graph Pad Software) was used for all statistical analysis. For comparison between two groups, a two-tailed unpaired *t* test was performed. For comparisons between more than two groups, a one-way ANOVA was performed, using either a Dunnett multiple comparisons test (compared with control condition) or Tukey multiple comparisons test (comparing all conditions with one another), unless otherwise noted. Groups were deemed statistically significantly different from one another if the *P* value was smaller than or equal to 0.05. On graphs, the symbols “*” and “#” were used to denote significance and defined as follows: *(#) *P* < 0.05; **(##) *P* < 0.01; ***(###) *P* < 0.001; ****(###) *P* < 0.0001. Comprehensive statistics readouts provided in the spreadsheet file labeled Supplementary Data S2—Statistical Analysis.

### Metabolomics Data Analysis

The QqQ data were preprocessed with Agilent MassHunter Workstation QqQ Quantitative Analysis Software (B0700). Each metabolite abundance level in each sample was divided by the median of all abundance levels across all samples for proper comparisons, statistical analyses, and visualizations among metabolites. The statistical significance test was done by a two-tailed *t* test with a significance threshold level of 0.10. All error bars represent mean with standard deviation. Heatmaps were generated and data clustered using Morpheus by Broad Institute (https://software.broadinstitute.org/morpheus). Pathway analyses were conducted using MetaboAnalyst (https://www.metaboanalyst.ca). Data available in Supplementary Data S3—Metabolomics.

### Data Availability

Data generated in this study are available as Supplementary files and/or spreadsheets, accompanied by legends. The three files are Supplementary File S1, S2, and S3 and respectively list all the proteomic, statistics, and metabolomic data that were generated in this study, which are complementary to the main provided text.

## Results

### CAF/CDM Functional Units Generate sEVs with Unique Protein Cargo

**FIGURE 1:**
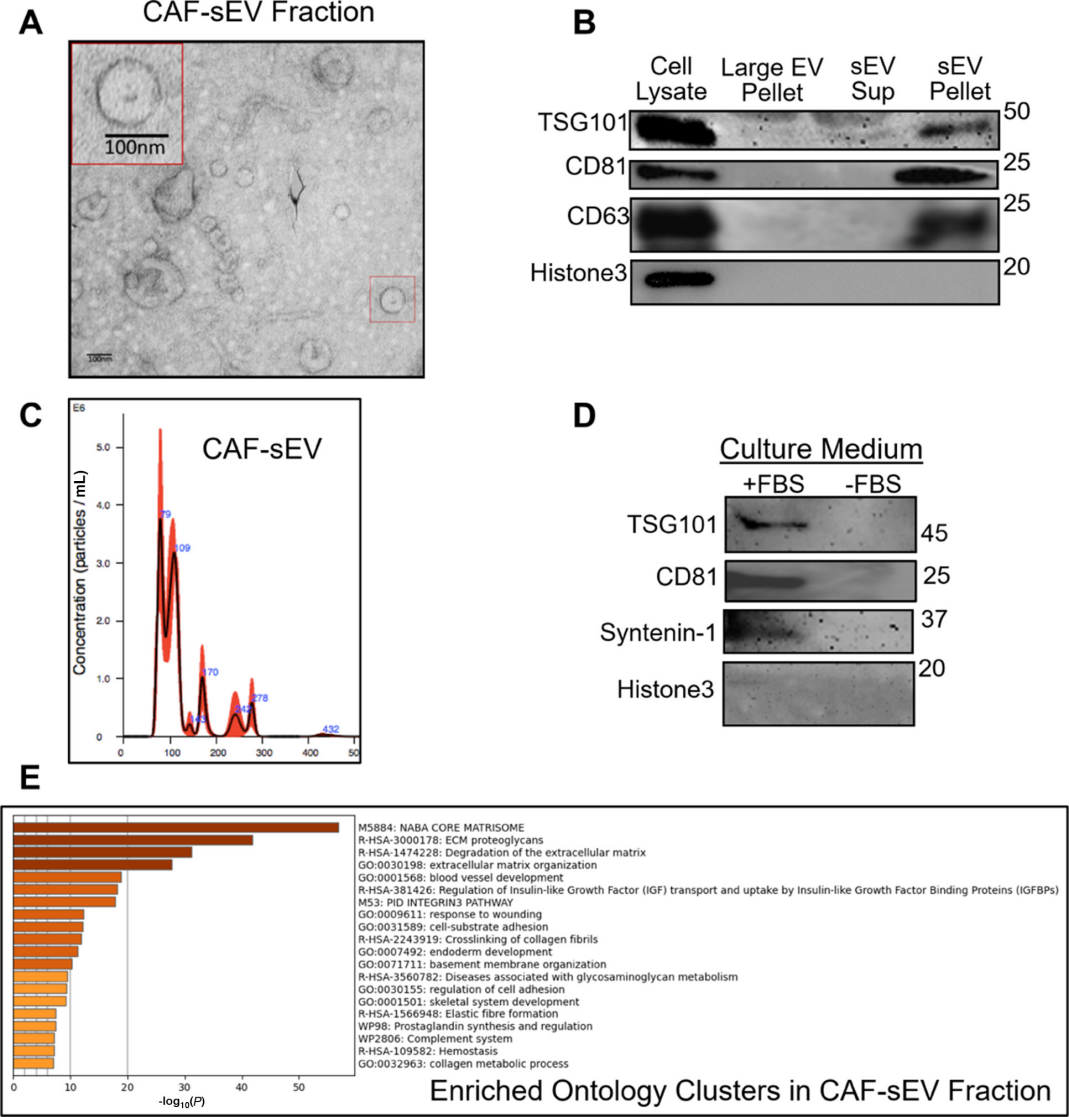
CDM-dependent CAFs generate sEVs with unique protein cargo. **A,** Representative transmission electron micrograph of sEVs isolated from CM collected from human pancreatic CAFs cultured within a CDM. Red box highlights a structure with canonical exosome morphology and size. Scale bar = 100 nm. **B,** Representative Western blot analysis of CAF lysate and assorted fractions collected following differential ultracentrifugation probing for enrichment of sEV markers. Lysate corresponding to 5 μg protein obtained from the EV-generating CAFs served as loading control. Fractions shown are: large EVs pellet (from the 10,000 × *g* centrifuge step); sEV supernatant; and sEV pelleted (collected from the 120,000 × *g* centrifuge step (see Materials and Methods for details). Note that the sEV pellet is enriched with canonical exosome markers and lacks organelle contaminants. **C,** Representative NTA histogram of an sEV pellet fraction; using the NanoSight platform. **D,** Control Western blot analysis showing sEVs isolated from media containing FBS (+FBS) and undetected in serum-depleted (−FBS) media. **E,** Enriched gene ontology clusters from proteomic analysis of CDM-producing human CAF isolated sEVs, *n* = 3. Note that full proteomics and enriched pathway analysis can be found in Supplementary Data S1—Proteomics (Tabs = Total proteins CAF.sEV, Fig. 1E Pathway Analysis)

We previously reported that tumor-supportive human CAFs, cultured within CDMs (e.g., CAF/CDM functional units), sustain PDAC cell survival under nutrient deprivation in a paracrine manner (27). To build on these findings, we investigated whether sEVs produced by tumor-supportive CAF units could account for the reported PDAC survival benefits. We focused on sEVs, as this subset is enriched with a biologically significant type of EV, exosomes, as well as other similarly sized vesicles (41, 42). Differential ultracentrifugation was used to isolate sEVs, and the resulting EV fraction was validated for the enrichment of exosomes according to standards dictated by the International Society for Extracellular Vesicles (43). Using transmitted electron microscopy, we observed that CAFs (27) generate heterogeneous sEVs that contain structures with canonical exosome morphology (Fig. 1A). Western blot analysis of CAF lysates and sequential sEV-isolation fractions indicated that the pelleted sEV fraction was enriched with canonical exosome markers, including TSG101, CD81, and CD63, and lacked nonspecific cellular fragment contaminants such as Histone 3 (Fig. 1B). This sEV enrichment was confirmed by nanoparticle tracking analysis (NTA), which showed that sEV particle sizes were in the appropriate range, concentrated in structures sized 80–180 nm (ref. 43; Fig. 1C). Technically, because serum is needed for CDM production and bovine EVs are present in the media used to generate the CAF/CDM functional units (e.g., complemented with FBS), CAFs were routinely switched to serum-free media following CDM production, assuring that sEVs harvested from CM and used in subsequent experiments were solely CAF-derived (Fig. 1D). To thoroughly validate the protein content of the CAF-sEV fraction, tandem mass spectrometry (e.g., MS/MS) analysis was performed, which identified a total of 226 proteins (Supplementary Data S1—Proteomics), highly enriched in known exosome-associated proteins. Using a cutoff of a minimum of five unique peptide sequences per protein, the top 60 proteins were used for a gene ontology query analysis, via Metascape (40). The most common cellular pathways identified were matrisome-related such as, integrin signaling, cell-substrate adhesion, insulin regulation, and wound healing (Fig. 1E; Supplementary Data S1—Proteomics). Together, these data serve as technical validation for the robust isolation of sEVs to be used in several subsequent assays, confirming their enrichment in exosomes, and reporting their unique CAF/CDM unit-derived protein signature; enriched in known exosomal biomarkers as expected, as well as in ECM proteins.

### NetG1^+^ CAFs Generate sEVs That Rescue PDAC Cells From Apoptosis Induced by Nutrient Deprivation

**FIGURE 2:**
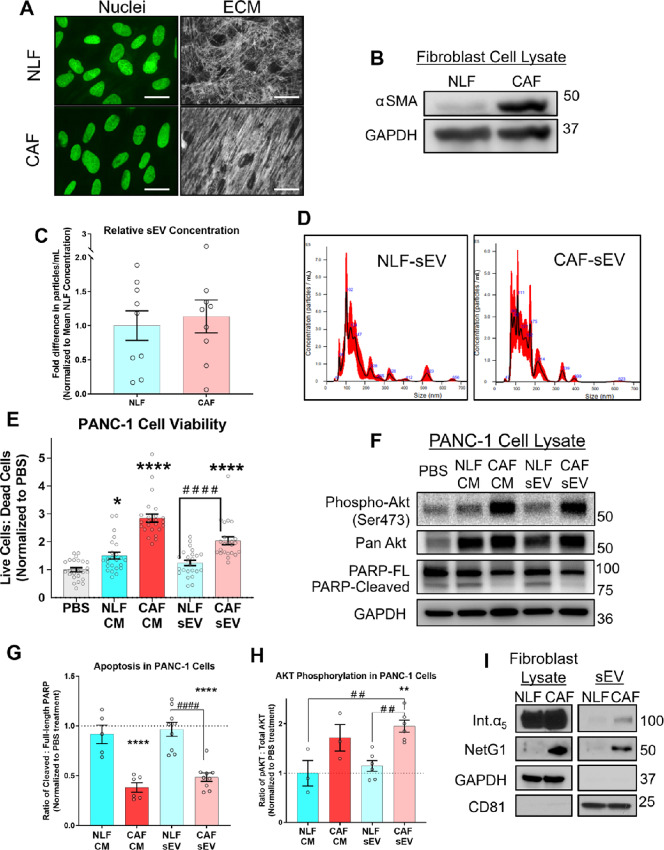
NetG1^+^ CAFs generate sEVs that rescue PDAC cells from nutrient deprivation-induced apoptosis. **A,** Representative confocal immunofluorescent images obtained from NLFs and CAFs cultured to produce a CDM. Shown are nuclei (SYBR green) and corresponding fibronectin ECM fibers (white). Scale bars = 50 μm; see Supplementary Fig. S1 for measurements. **B,** Representative Western blot analysis of CAF and NLF cell lysates, probing for expression of myofibroblastic activation marker, αSMA. GAPDH as protein loading control. **C,** Relative differences in total particle concentrations found in sEV fractions isolated from NLFs and CAFs, normalized to the mean NLF value; using the NanoSight platform. Bars = standard error. Statistics: Unpaired *t* test using Welch correction. *n* = 4. Comprehensive statistical readouts provided in Supplementary Data S2—Statistical Analysis (Tab = Fig. 2C). **D,** Representative NTA histograms of sEV fractions from NLF and CAFs, using the NanoSight platform. **E,** Cell viability assay of PANC-1 cells at 48 hours posttreatment with CM or sEV from NLFs and CAFs. *n* = 3 biological replicates; each replicate consists of six technical repeats. All repeats per replicate were normalized to the mean of the corresponding PBS-treated condition. Bars = standard error. Statistics: one-way ANOVA, with multiple comparisons using Tukey correction. *, Compared with PBS (negative control), # comparing between conditions noted by connecting lines. A comprehensive list of statistical readouts is provided in Supplementary Data S2—Statistical Analysis (Tabs = Fig. 2E). **F,** Representative Western blots of PANC-1 cell lysates collected 48 hours posttreatment with PBS, CM, or sEVs from NLF or CAF cells. Probing for Akt activation via phosphorylation at Serine 473 and apoptosis occurring via PARP cleavage (lower band). Pan Akt as pAkt control, GAPDH as protein loading control. **G,** Apoptosis occurring in PANC-1 cells as measured by the ratio of cleaved PARP: full-length PARP, quantified from the optical density of digitized Western blots in **F** using the software ImageJ. *n* = 6. **H,** Akt activation as measured via the ratio of phosphorylated Akt at Serine 473: Pan Akt, quantified from the optical density of digitized Western blots in **F** using the software ImageJ. *n* = 3. **G** and **H,** Results were normalized to the PBS-treated condition (negative control) for each replicate blot, represented by dotted line. Bars = standard error. Statistics: one-way ANOVA, with multiple comparisons using Tukey correction. * Compared with PBS, # comparing between conditions noted by connecting lines. A comprehensive list of statistical readouts is provided in Supplementary Data S2—Statistical Analysis (Tabs = Fig. 2G, 2H). **I,** Representative Western blots of NLF and CAF cell lysate and sEV fractions, probing for NetG1 and Int.α_5_. GAPDH as protein loading control in cell lysate fraction. CD81 as loading control in sEV fraction.

Well-characterized pancreatic human NLFs and CAFs were used throughout this study (27–29). Still, we validated their established phenotypes through a set of quality control assays. First, the architecture of their CDMs were tested to confirm that CAFs produce the characteristic dense and anisotropic (e.g., aligned) CDMs, compared with the isotropic (e.g., disorganized) CDMs produced by NLFs (Fig. 2A; Supplementary Fig. S1). Next, we also confirmed that NLFs do not express the canonical contractile fibroblastic marker αSMA (44), known to be artificially induced in classically-cultured (e.g., in a monolayer) “normal” fibroblastic cells (Fig. 2B). Furthermore, CAFs were found to be more tumor-supportive than NLFs when in direct coculture with PDAC cells under nutrient deprivation, similarly to our previous studies (ref. 27; Supplementary Fig. S2). Of note, these phenotypic analyses were conducted to assure that sEVs used in all experiments were collected from fibroblastic cells that sustained the reported traits when cultured *in vitro* (29, 30).

Upon initial analysis of sEVs collected from CAFs versus NLFs, we noted no significant differences in amounts of total sEVs secreted by these cells, while minor changes in sEV size distributions were apparent (Fig. 2C and D). Furthermore, to determine whether sEVs isolated from NetG1^+^ CAF-CM retain tumor-supportive functions, we tested the ability of CAF-sEVs to support human PDAC cells cultured under poor nutrient conditions (e.g., serum and glutamine free). Similar to CAF-generated CM, for which an obvious survival benefit was observed at 48 hours (Supplementary Fig. S3A), CAF-derived sEVs were about 2-fold more effective than NLFs-derived sEVs in providing a survival benefit to nutrient-deprived PDAC cells (Fig. 2E; Supplementary Fig. S3B–E). This CAF-sEV mediated survival benefit was likely due to the inhibition of apoptosis, as Western blot probing of PARP showed there was significantly less PARP cleavage, a downstream indicator of apoptosis induction (45), in nutrient-deprived PDAC cells treated with CAF-sEVs compared with NLF-sEVs (Fig. 2F–G; Supplementary Fig. S3F). In addition, we found that Akt phosphorylation was also increased in PDAC cells treated with CAF-sEVs compared with those treated with NLF-sEVs (Fig. 2F–H), suggesting active signal transduction occurring in prosurvival pathways (46, 47). Finally, we probed for two CAF-associated protein markers, NetG1 and Int.α_5_ (27, 28), to develop a better understanding of the differences between the sEVs derived from CAF versus NLF, which could account for the functional results. Western blot analysis revealed an enrichment in both NetG1 and Int.α_5_ in CAF-sEVs as compared with NLF-sEVs (Fig. 2I), illustrating differential expression of two critical CAF markers that can dictate their phenotypes. Taken together, these results demonstrate that CAF-sEVs contain a unique tumor-supportive phenotype compared with NLF-sEVs, and are capable of stimulating signal transduction in Akt-mediated survival pathways in PDAC cells, which in turn prevents PDAC cells from undergoing apoptosis during states of nutritional stress.

### NetG1^+^ CAF-sEVs Support PDAC Cell Survival in an NGL-1–dependent Manner

The sole known heterotypic receptor for NetG1 is the postsynaptic transmembrane protein NetG1-ligand (NGL-1; refs. 48, 49). We recently reported that NGL-1 is expressed in human and murine PDAC tissue, and that its expression is needed for the nutritional benefit that is imparted by NetG1^+^ CAFs *in vitro* and *in vivo* (27). Thus, we queried the levels of NGL-1 expression in a panel of human pancreatic tumorigenic cells. Results confirmed that NGL-1 levels in these malignant cells are significantly higher than levels detected in benign HPNE cells (ref. 31; Supplementary Fig. S4A). Therefore, we posited a role for the NetG1/NGL-1 axis in the observed tumor-supportive function of NetG1^+^ CAF-EVs. To test this, we generated NGL-1 knockdown (KD) PANC-1 cells (Supplementary Fig. S4B; ref. 27), and treated these with NetG1^+^ CAF-sEVs as done previously. Baseline survival during nutrient-deprived conditions showed that NGL-1^KD^ cells have a tendency for poorer survival compared with control (Ctl.) PANC-1 cells. More importantly however, the CAF-sEV–mediated survival benefit seen in Ctl. PDAC cells was abolished in NGL-1^KD^ PDAC cells treated with the same sEVs (Supplementary Fig. S4C), demonstrating that an NGL-1 deficiency in PDAC cells prevents the tumor-supportive benefit imparted by NetG1^+^ CAF-generated sEVs. To further test the involvement of NetG1/NGL-1 in this system, we preincubated NetG1^+^ CAF-sEVs with increasing concentrations of recombinant NGL-1 (rNGL-1) and assessed whether the soluble protein could play an antagonistic role in this system by preventing the observed survival benefit imparted by the untreated sEVs (Supplementary Fig. S4D). Excitingly, we observed that while rNGL-1 produced no observable cytotoxicity on its own, if incubated with CAF-sEVs, increasing amounts of rNGL-1 resulted in a significant decrease in PDAC cell survival under nutrient-deprived conditions (Supplementary Fig. S4E). Together, these data suggest that disruption of the NetG1/NGL-1 axis reduces the ability for CAF-sEVs to improve the survival of nutrient-deprived PDAC cells.

### NetG1 Expression in CAFs is Necessary for the sEV-mediated Survival of Nutrient-deprived PDAC Cells

**FIGURE 3:**
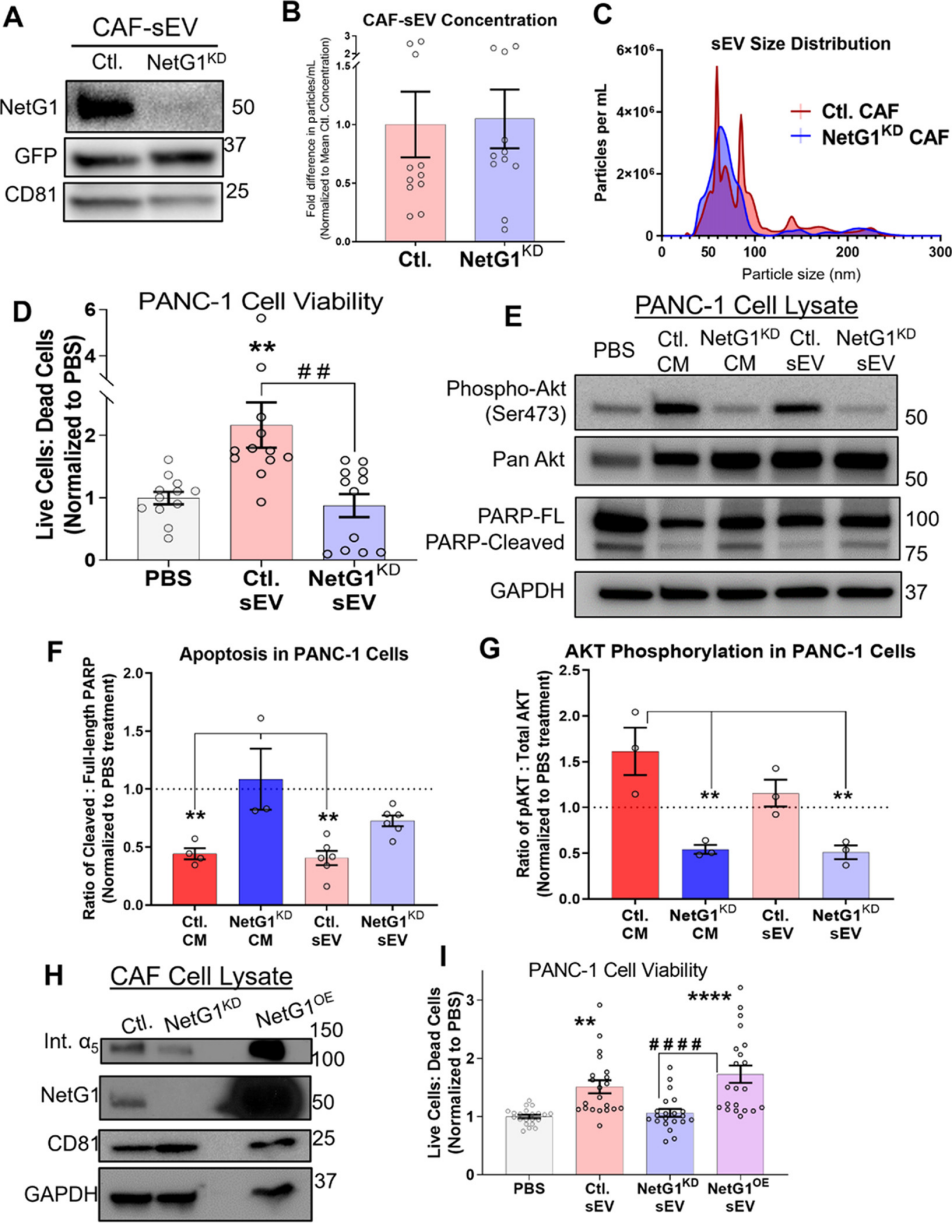
NetG1 expression in CAFs is necessary for the sEV-mediated survival of nutrient-deprived PDAC cells. **A,** Representative Western blots of CAF-sEV lysates showing effective ablation of NetG1 in sEVs from NetG1^KD^ CAFs. GFP and CD81 as loading controls. **B,** Relative differences in total particle concentrations found in sEV fractions isolated from Ctl. versus NetG1^KD^ CAFs, normalized to the mean Ctl. CAF-sEV value. Bars = standard error. Statistics: Unpaired *t* test using Welch correction. *n* = 4. Comprehensive statistical readouts provided in Supplementary Data S2—Statistical Analysis (Tab = Fig. 3B). **C,** Histograms of nanoparticle distribution of sEV fractions from Ctl. versus NetG1^KD^ CAFs sEV fractions (arbitrarily cutoff at 300 nm), superimposed to show differences in EV populations; using the NanoSight platform. **D,** Cell viability assay of PANC-1 cells at 48 hours posttreatment with sEVs from Ctl. or NetG1^KD^ CAFs. *n* = 3 biological replicates. **E,** Representative western blots of PANC-1 cell lysates collected 48 hours posttreatment with CM, or sEVs from Ctl. or NetG1^KD^ CAFs. Probing for Akt activation via phosphorylat—ion at Serine 473 and apoptosis occurring via PARP cleavage (lower band). Pan Akt as phosho-Akt control, GAPDH as protein loading control. **F,** Apoptosis occurring in PANC-1 cells as measured by the ratio of cleaved PARP: full-length PARP, quantified from the optical density of digitized Western blots in Fig. 2F using the software ImageJ. *n*≥ 3. **G,** Akt activation as measured via the ratio of phosphorylated Akt at Serine 473: Pan Akt, quantified from the optical density of digitized Western blots in Fig. 3E using the software ImageJ. *n* ≥ 3. **F and G,** Results were normalized to the PBS-treated condition (negative control) for each replicate blot; represented by dotted line. Bars = standard error. Statistics: one-way ANOVA, with multiple comparisons using Tukey correction. * Comparing between conditions noted by connecting lines. Comprehensive statistical readouts provided Supplementary Data S2—Statistical Analysis (Tabs = Fig. 3F, 3G). **H,** Representative Western blot analysis of CAF cell lysates, validating the levels of expression of the indicated markers in Ctl., NetG1^KD^, and NetG1^KD^ CAFs reexpressing ectopic NetG1 (NetG1^OE^). GAPDH as protein loading control. **I,** Cell viability assay of PANC-1 cells at 48 hours posttreatment with sEVs from Ctl., NetG1^KD^, and NetG1^OE^ CAFs. *n* = 3 biological replicates. For **D** and **I**: Each replicate consists of six technical repeats. All repeats per replicate were normalized to the mean of the corresponding PBS-treated condition. Bars = standard error. Statistics: one-way ANOVA, with multiple comparisons using Tukey correction. * Compared with PBS (negative control), # comparing between conditions noted by connecting lines. Comprehensive statistical readouts provided in Supplementary Data S2—Statistical Analysis (Tabs = Fig. 3D, 3I).

Next, we aimed to shift our focus to NetG1’s role in the ability of CAFs to produce tumor-supportive sEVs. To achieve this, we employed clustered regularly interspaced short palindromic repeats’ interference (CRISPRi)-generated NetG1^KD^ CAFs which have been characterized previously (27). Ctl. CAFs used included an empty CRISPRi vector, and both CAF lines were engineered to express GFP, allowing us to track the transfer of “nonspecific cargo” (Fig. 3A). NTA data demonstrated that NetG1^KD^ CAFs produce a similar amount of sEVs compared with Ctl. CAFs (Fig. 3B), suggesting NetG1 ablation does not result in a dysfunction in sEV biogenesis and/or secretion. Of note, we observed differences in mean particle size distributions (Fig. 3C), alluding to a possible role for NetG1 in modulating the type of EVs and/or EV cargo generated by CAFs. When tested functionally, we observed that sEVs from NetG1^KD^ CAFs did not produce a prosurvival benefit to nutrient-deprived PDAC cells (Fig. 3D; Supplementary Fig. S5A), simulating the effects observed by sEVs from NLFs (Fig. 2; Supplementary Fig. S3). This tumor-restrictive behavior was further emphasized by the increased PARP cleavage and decreased Akt phosphorylation in PANC-1 cells treated with NetG1^KD^ CAF-sEVs compared with Ctl. CAF-sEVs (Fig. 3E–G). These results demonstrate that NetG1 ablation in CAFs effectively phenocopies NLFs, in that their secreted sEVs cannot stimulate prosurvival signaling capable of preventing starvation-induced apoptosis in PDAC cells.

To further validate the tumor-supportive role that NetG1 plays in CAF-sEVs, we overexpressed (OE) NetG1 in NetG1^KD^ CAFs (NetG1^OE^ CAF). NetG1 overexpression was confirmed by Western blot analysis (Fig. 3H). Similar to NetG1^KD^, NetG1^OE^ did not change total sEV numbers; however, the sEV size distribution was again slightly shifted (Supplementary Fig. S5B). Importantly, the reintroduction of NetG1 effectively reinstituted the ability of sEVs to provide a significant survival benefit to PDAC cells cultured under nutrient-deprived conditions (Fig. 3I; Supplementary Fig. S5C), as survival levels observed returned to those conferred by Ctl. CAF-sEVs. Taken together, these data demonstrate that CAFs require NetG1 expression to produce sEVs capable of successfully supporting the survival of PDAC cells under nutrient deprivation.

### Loss of NetG1 in CAFs Alters the Metabolite Profile of CAF-sEVs

We next aimed to characterize the NetG1-dependent cargo of CAF-sEVs. To first determine whether NetG1 expression in CAFs affected sEV uptake by PDAC cells, we tracked GFP transferred as cargo in CAF-sEVs to recipient PDAC cells. Interestingly, we observed a similar amount of GFP to be found in PDAC cell lysates treated with sEVs from either Ctl. or NetG1^KD^ CAFs (Supplementary Fig. S6A and S6B), which suggested that uptake of sEVs generated from CAFs deficient in NetG1 was not impaired and was likely not responsible for the differences observed in PDAC survival.

**FIGURE 4:**
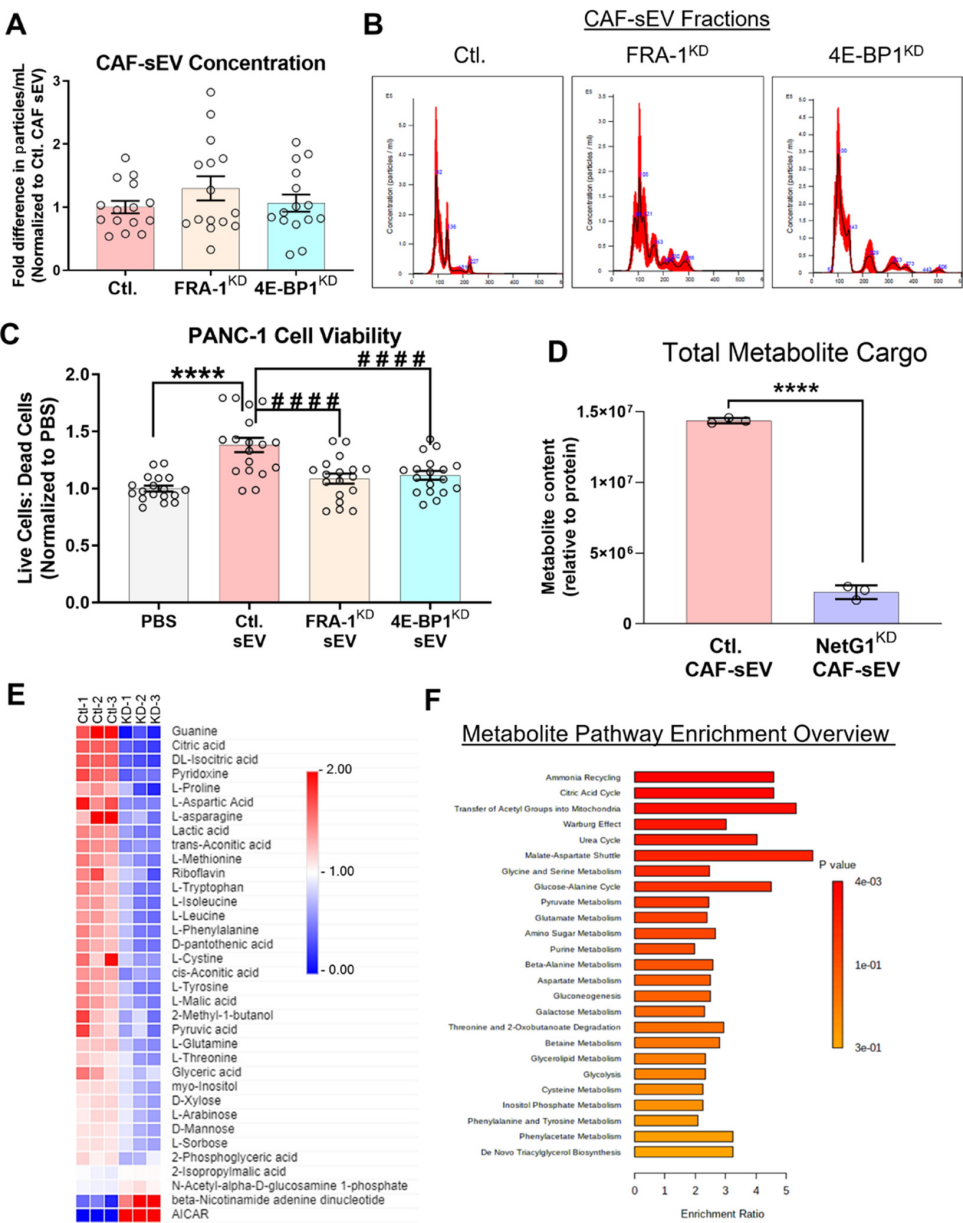
Loss of NetG1 in CAFs alters the metabolite profile of CAF-sEVs. **A,** Relative differences in total particle concentrations found in sEV fractions isolated from Ctl. versus FRA1^KD^, and 4EBP1^KD^ CAFs; normalized to the mean Ctl. CAF-sEV value. Bars = standard error. Statistics: one-way ANOVA, with multiple comparisons using Tukey correction. *n* = 4. Comprehensive statistical readouts provided in Supplementary Data S2—Statistical Analysis (Tab = Fig. 4A). **B,** Representative NTA histograms of sEV fractions from Ctl., FRA1^KD^, and 4EBP1^KD^ CAFs; using the NanoSight platform. **C,** Cell viability assay of PANC-1 cells at 48 hours posttreatment with sEVs from Ctl., FRA1^KD^, or 4EBP1^KD^ CAFs. *n* = 3 biological replicates; each replicate consists of 6 technical repeats. All repeats per replicate were normalized to the mean of the corresponding PBS-treated condition. Bars = standard error. Statistics: one-way ANOVA, with multiple comparisons using Tukey correction. * compared with PBS (negative control), # comparing between conditions. Comprehensive statistical readouts provided in the Supplementary Data S2—Statistical Analysis (Tab = Fig. 4C). **D,** Total amount of metabolites detected in sEVs isolated from Ctl. versus NetG1^KD^ CAFs, normalized to equal protein levels. *n* = 3. Bars = standard error. Statistics = unpaired *t* test using Welch correction. Comprehensive statistical readouts provided in Supplementary Data S2—Statistical Analysis (Tab = Fig. 4D). **E,** Heatmap depicting significant metabolite differences in sEVs isolated from control Ctl. versus NetG1^KD^ CAFs, normalized to median sEV metabolite levels. A *t*-test *P* value of <0.01 was used as a cutoff threshold. A full readout of metabolomic analysis can be found in Supplementary Data S3—Metabolomics (Tabs = Ctl SEV vs. NetG1KD sEV, Ctl SEV vs. NetG1KD SEV HM). **F,** Pathway enrichment analysis summary generated by MetaboAnalyst using metabolomics data in **E**. The top 25 most significantly represented pathways are shown, ranked *P* value in descending order of significance.

We previously reported that NetG1-modulated CAF metabolism accounts for the tumor-supportive qualities of CAF-CM during nutritional stress (27). This effect was found to be partially modulated by two pathways regulated downstream of NetG1, P38 represented by FRA-1 and Akt by 4E-BP1, whose ablation in CAFs produced a downregulation in glutamine (Gln) secretion. To build upon these findings, sEVs were isolated from previously characterized FRA-1^KD^ and 4E-BP1^KD^ CAFs and their tumor-supportive capabilities were assessed. While there were no significant differences in the amount of sEVs secreted by these CAFs (Fig. 4A and B), sEVs from FRA-1^KD^ and 4E-BP1^KD^ CAFs were unable to provide prosurvival benefits to nutrient-deprived PDAC cells compared with those from Ctl. CAFs (Fig. 4C). These results were similar to that of sEVs from NetG1^KD^ CAFs (Fig. 3), and thus it was postulated that the loss of function observed in the sEVs could be due the dysregulation of NetG1-modulated CAF metabolism.

These findings justified a more comprehensive dissection of the metabolite profiles in Ctl. versus NetG1^KD^ CAF-sEVs, which was performed via metabolomic analysis. When total metabolite content in sEVs was normalized to equal levels of protein, it was found that NetG1^KD^ CAFs produce sEVs with approximately 6-fold less metabolites compared to Ctl. CAFs (Fig. 4D), thus confirming a NetG1-dependent regulation of sEV metabolite loading. Furthermore, specific metabolites were significantly enriched in Ctl. CAF-sEVs; one such being Gln (Fig. 4E). In addition, pathway enrichment analyses highlighted that upon NetG1 loss, CAFs generate sEVs with cargo deregulating numerous key metabolic pathways such as, ammonia recycling, citric acid cycle, transfer of acetyl groups into mitochondria, the Warburg effect, the urea cycle, and others (Fig. 4F). Altogether, these results demonstrate that NetG1 expression in CAFs regulates the metabolic content of sEVs.

### NetG1 is Found in Distinct Nanoparticles That Differ From Int.α_5_^+^ Exosomes, and Both Contribute to PDAC Cell Survival Under Nutrient Deprivation

**FIGURE 5:**
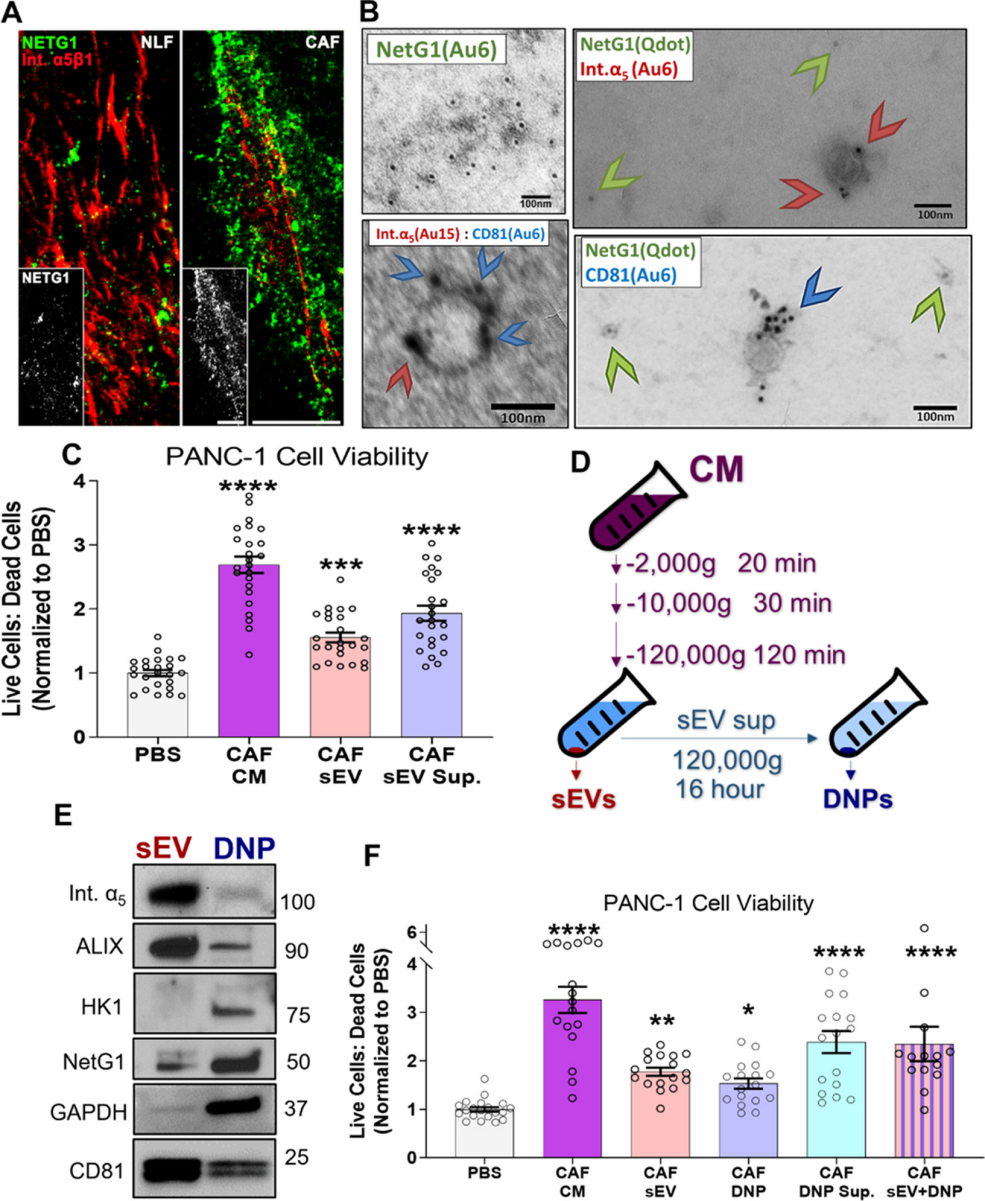
NetG1 is found in distinct nanoparticles that differ from Int.α_5_^+^ exosomes and both support PDAC cell survival under nutrient deprivation. **A,** Representative confocal images of the extracellular surface of the plasma membrane of an NLFs (left) and CAFs (right) immunofluorescently labeled with Int.α_5_β_1_ (red) and NetG1 (green) tags. NLFs and CAFs cultured in their respective CDMs and fixed/labeled under nonpermeable conditions to prevent labeling of intracellular compartments. Inserts are monochromatic images of NetG1 (white). Scale bars = 10 μmol/L. **B,** Representative TEM images of immunolabeled CAF sEVs. Top left, Immunolabeling of NetG1 with anti-NetG1 antibody-conjugated gold particles (black dots), Top right, Double immunodetection of NetG1 and CD81 using anti-NetG1 antibody-conjugated quantum dot (low-density dots; green arrowheads) and anti-CD81 antibody-conjugated gold particles (high-density dots; red arrowheads), (bottom left) double immune detection of CD81 (small dots pointed by blue arrowheads) and activated Int.α5 using SNAKA51-conjugated gold particles (big dots pointed by red arrowheads). Bottom right, Double immunodetection of NetG1 and activated Int.α5 using anti-NetG1 antibody-conjugated quantum dot (low-density dots; green arrowheads) and SNAKA51-conjugated gold particles (high-density dots; blue arrowheads). Scale bars = 100 nm. **C,** Cell viability assay of PANC-1 cells at 48 hours posttreatment with the indicated fractions of CAF-secreted material. *n* = 3 biological replicates. **D,** Schematic describing the modified differential ultracentrifugation technique used to isolate DNPs from sEV supernatant. **E,** Representative Western blot analysis of CAF-derived sEV and DNP fractions depicting NetG1 enrichment in DNPs and Int.α5 enrichment in sEVs. CD81, ALIX as positive sEV controls; hexokinase1 (HK1), GAPDH as positive DNP controls. **F,** Cell viability assay of PANC-1 cells at 48 hours posttreatment with the indicated fractions of CAF-secreted material. *n* = 4 biological replicates. For **C** and **F**: each biological replicate consists of six technical repeats. All repeats per replicate were normalized to the mean of the corresponding PBS-treated condition. Bars = standard error. Statistics: one-way ANOVA, with multiple comparisons using Tukey correction. * Compared with PBS (negative control). Comprehensive statistical readouts provided in the Supplementary Data S2—Statistical Analysis (Tabs = Fig. 5C, 5F).

To develop a better characterization of the EVs secreted by NetG1^+^ CAFs, we next turned our focus to dissecting the unique subpopulations of EVs. The type of EV that a given protein can be found in will be dictated by its subcellular localization, where proteins located at the PM are likely to be secreted in EVs that are distinct from those presenting proteins that trafficked intracellularly via the endosome (50, 51). This is relevant to our investigation as we previously reported that the localization of active Int.α_5_ changes from being at the PM in NLFs to intracellular multivesicular bodies in CAFs (28), and herein we noted that NetG1 is expressed at the PM in CAFs (Fig. 5A). Importantly, we understand our sEV population to be heterogeneous (Fig. 1), and as NetG1 and Int.α_5_ have differential subcellular localization in CAFs, we hypothesized that these proteins may be incorporated in unique populations of EVs. To test this, we immuno-labeled sEVs using gold or quantum dot–conjugated primary antibodies. CD81 was used as a canonical exosome biomarker and its localization was compared with that of NetG1 and the active conformation of Int.α_5_. Interestingly, NetG1 was detected in DNPs characteristic of exomeres (22, 23), spanning about 20 nm in diameter (Fig. 5B). These NetG1^+^ EVs were distinct from classical exosomes in morphology, and did not colocalize with CD81^+^ vesicles. In contrast, EVs that were positive for Int.α_5_β_1_ were exosome-like in morphology and size, and colocalized with CD81, suggesting Int.α_5_ is secreted in exosomes. Importantly, NetG1 and Int.α_5_ were not observed to colocalize to the same EVs, supporting our hypothesis that their differential cellular localization in CAFs would confer their loading into unique EVs.

These results prompted us to investigate the contributions of sEVs and DNPs in greater detail. Important to note, sEV fractions were isolated using differential ultracentrifugation, which separates particles based on density and size (52). As NetG1^+^ EVs were observed to be smaller than exosomes (e.g., DNPs), it was hypothesized that a significant proportion of these DNPs may be lost when discarding the supernatant of the sEV pellet, which is validated primarily for its enrichment of exosomes (33). NTA data of these two CAF fractions confirmed that the sEV supernatant was indeed enriched in smaller EVs as compared with the sEV pellet (Supplementary Fig. S7A), and moreover this supernatant fraction contained tumor-supportive properties (Fig. 5C). As previous studies have identified that exomeres and exosomes carry distinct cargo (23, 34), we postulated that DNPs could also serve a functional role in PDAC survival. To further fractionate subpopulations of EVs from CAF-CM, we employed a modified version of the differential ultracentrifugation protocol (34), reported to effectively precipitate DNPs from sEV supernatants (Fig. 5D). We confirmed via NTA that this protocol was effective in isolating EVs that were enriched in smaller particles than that in the sEV fraction (Supplementary Fig. S7B), thus providing us with two unique EV fractions to assay: sEVs enriched in exosomes, and DNPs enriched in exomeres. Western blot analysis of these fractions confirmed our previous EM data (Fig. 5B), demonstrating an enrichment of NetG1 in the DNP fraction, accompanied by a decrease in Int.α_5_ and exosome markers ALIX and CD81 (Fig. 5E). In addition, and as expected, we confirmed previous reports that glycolysis enzymes hexokinase-1 and GAPDH are increased in DNPs (refs. 22, 34; Fig. 5E). Importantly, the NetG1-enriched DNP fraction was also functionally significant in providing a survival benefit to nutrient-deprived PDAC cells (Fig. 5F). Together, these results characterize a novel NetG1^+^ EV subpopulation with tumor-supportive properties, as well support our hypothesis that NetG1 and Int.α_5_β_1_ are loaded into distinct EVs based on differential subcellular localization in CAFs.

### CAF-sEV and CAF-DNP Subpopulations Have Distinct Proteomic and Metabolomic Profiles

**FIGURE 6:**
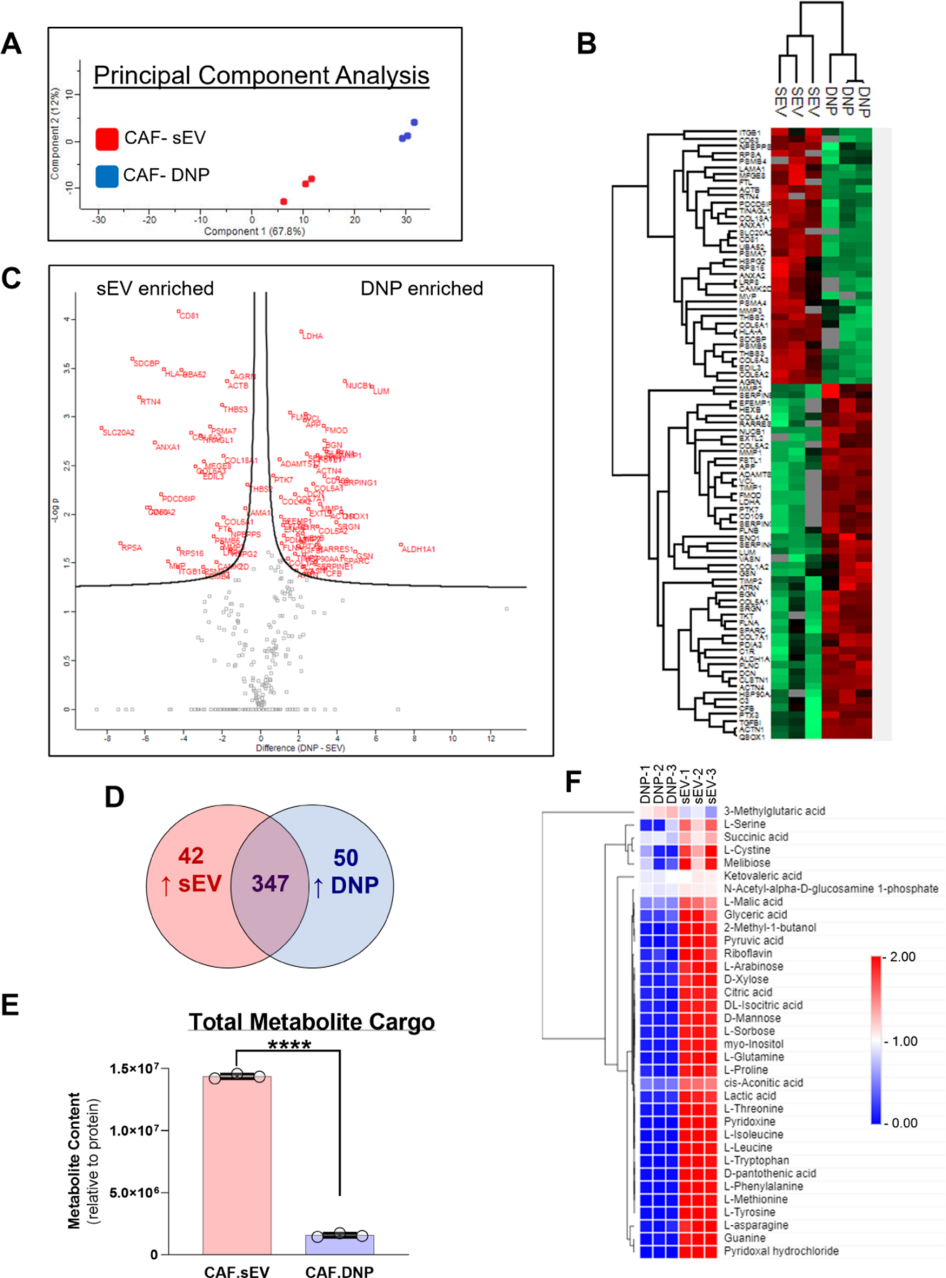
Proteomic and metabolomic profiling distinguish between CAF-EV subpopulations. **A,** Principal component analysis of CAF-derived sEV and DNP replicate samples. Tight clustering of replicates indicates reliable reproducibility in sample integrity and processing. **B,** Heatmap visualizing protein enrichment in individual replicates of sEV and DNP fractions. **C,** Volcano Plot comparing individual protein levels in sEV versus DNP fractions. Red labels indicate statistically significant proteins enriched in sEV (left side) or in DNP (right side) fractions. Full readout of proteomic analysis can be found in Supplementary Data S1—Proteomics (Tab = Fig. 6C, Volcano plot signatures). **D,** Venn diagram visualizing the numbers of unique proteins enriched in sEV or DNP fractions. Parameters for Student *t* test were as follows: S0 = 2, side both using Benjamini–Hochberg discovery rate <0.05. A full list of proteins enriched in each fraction can be found in Supplementary Data S1—Proteomics (Tab = sEV and DNP protein enrichments). **E,** Total amount of metabolites detected in CAF-derived sEVs versus DNPs, normalized to equal protein levels. *n* = 3. Bars = standard error. Statistics = unpaired *t* test using Welch correction. Comprehensive statistical readouts provided in the Supplementary Data S2—Statistical Analysis (Tab = Fig. 6E). **F,** Heatmap depicting CAF-derived sEV versus DNPs, normalized to median EV metabolite levels. Shown are the metabolites that differed significantly between sEVs and DNPs using a *t*-test *P* value of <0.01. A full readout of metabolomic analysis can be found in Supplementary Data S3—Metabolomics (Tabs = Ctl DNP vs. Ctl sEV; Ctl DNP vs. Ctl SEV HM).

While both CAF-derived EV fractions have tumor-supportive capabilities, their differences in size and markers suggest that their modes of biogenesis may confer different protein and metabolite profiles. To better characterize the differences in protein composition between CAF-secreted sEVs and DNPs, we conducted a LFQ proteomic profiling (53). Principal component analysis of the sample replicates indicated that sEV and DNP fractions corresponded to the enrichment of two distinct populations, with a high degree of consistency within experimental conditions (Fig. 6A). The relative protein differences between the two EV types were visualized via a heatmap, of which the most significantly enriched proteins in each fraction were identified; constituting 42 proteins uniquely represented in sEVs and 50 in DNPs (Fig. 6B–D). As expected and consistent with our previous results, the sEV fraction was significantly enriched in exosome markers such as CD81, CD63, ALIX (PDCD6IP), and Syntenin1 (SDCBP). This fraction also included β1-integrins (known to form heterodimers with Int.α_5_), as well as HLA-class1 histocompatibility antigen, and β-actin, consistent with previous reports (33). Furthermore, the DNP fraction was enriched with proteins relating to glycolysis and other metabolic pathways upregulated in cancer, including lactate dehydrogenase A (LDHA) (54), transketolase (55), and β-hexosaminidase (56). Notably, β-hexosaminidase has also been reported in exomeres (23). Together, these data demonstrate robust proteomic differences in the composition of CAF-derived sEVs and DNPs.

Next, using Metascape's gene annotation and analysis resource (40), we identified a number of signaling pathways unique to each fraction (Supplementary Fig. S8A). In addition, metabolomic analyses comparing sEVs and DNPs (normalized to total EV protein), indicated that CAF-sEVs have 9-fold higher metabolite cargo compared with CAF-DNPs (Fig. 6E). When querying specific metabolite differences (normalized to median metabolite levels), sEVs were enriched in protumor metabolites such as Gln and proline, while only 3-methylglutaric acid was enriched in DNPs (Fig. 6F). Of note, pathway enrichment analysis of metabolites enriched in sEVs compared with DNPs identified several anabolic pathways important in cell survival and proliferation (refs. 57–59; Supplementary Fig. S8B). In addition, rupturing the membranes in both fractions, via boiling followed by filtering of protein aggregates, significantly reduced the tumor-supportive effects of the EVs (Supplementary Fig. S9). This indicated that irrespective of EV origin and packaging (i.e., exosome vs. exomere; MVB vs. PM), boiling disrupts the EV-dependent survival benefits to PDAC cells under nutrient-poor conditions. Altogether, these results serve to further characterize CAF-derived sEV and DNP fractions by identifying distinctive protein and metabolite profiles in each of these EV subpopulations.

### NetG1 and Int.α_5_ are Enriched in sEVs Isolated from Plasma of Patients with PDAC

**FIGURE 7:**
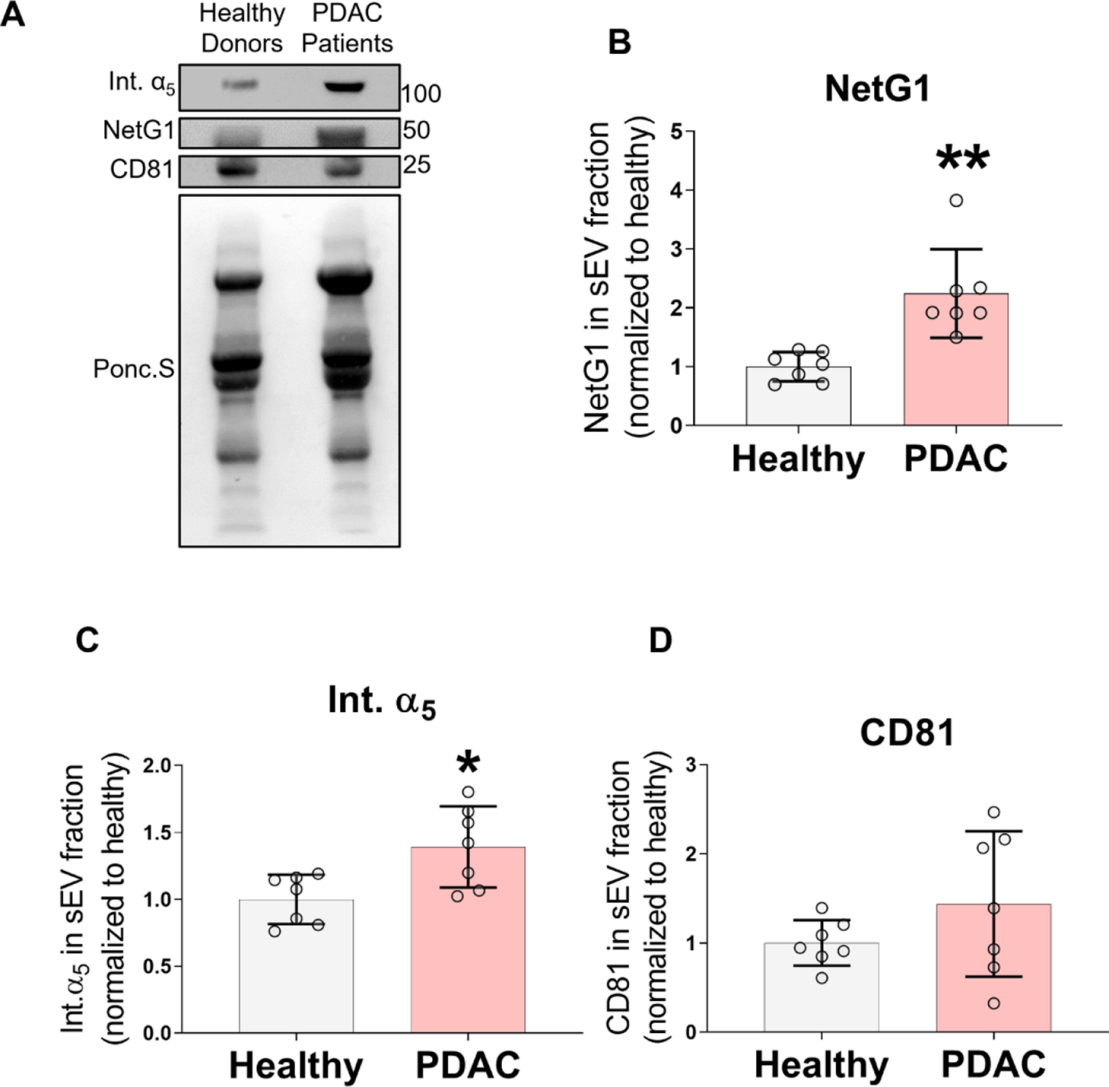
NetG1 and Int.α_5_ are enriched in sEVs isolated from plasma of patients with PDAC**. A,** Representative Western blots of sEV fractions collected from plasma of human patients with PDAC (*n* = 6) and healthy age/sex-matched control subjects (*n* = 4). Ponceau S stain as total protein loading control. **B–D,** Quantification of all blots represented in **A**. Levels of NetG1 (**B**), Int.α_5_ (**C**), and CD81(**D**) levels in sEVs were divided by their total protein (Ponceau S) and the results were normalized to the mean healthy value. Bars = standard error. Statistical test used: Unpaired *t* test, with Welch correction. Comprehensive statistical readouts provided in the Supplementary Data S2—Statistical Analysis (Tabs = Fig. 7B, 7C, 7D).

Finally, we wanted to determine whether the CAF-sEV profile we identified earlier was represented in a clinical setting. Because CAFs comprise such a significant proportion of the cellular content in PDAC tumor masses, we posited that sEVs collected from human PDAC patient plasma could be enriched with pancreatic CAF markers identified earlier in this study. Thus, we probed for levels of NetG1 and Int.α_5_ in sEVs isolated from human PDAC patient plasma and compared these with levels detected in sex and age matched healthy volunteers’ plasma. Encouragingly, we observed a 2.2- and 1.4-fold increase in NetG1 and Int.α_5_ levels, respectively in sEVs from patients with PDAC compared with those from healthy control samples (Fig. 7A–C). Importantly, we did not detect significant differences in the amount of the canonical exosome marker CD81 that was detected across all samples (Fig. 7D). Together, these results recapitulate our *in vitro* data and provide evidence that NetG1 and Int.α_5_ could serve, in the future, as potential circulating biomarkers indicative of a pancreatic stromal state supportive of PDAC in human subjects.

## Discussion

This investigation sought to shed light on the relationship that exists between PDAC cancer cells and their TME, specifically CAFs. During PDAC development, the pancreatic TME becomes characteristically desmoplastic and nutrient-poor, creating physiologically stressful state for cancer cells. Previous studies published by our group have shown that CAFs are able to aid PDAC cells in overcoming these states of nutrient deprivation promoting their survival and disease progression (27). Furthermore, this tumor-supportive behavior displayed by CAFs is performed in a paracrine manner, as CAFs secrete material into the extracellular environment, capable of modulating the survival of PDAC cells. Our group has previously identified several protumor protein and metabolite factors secreted by CAFs, and moreover determined that tumor-supportive properties of this CAF-secreted material is regulated, in part, by the CAF expression of NetG1 (27). Our current study builds upon these discoveries, aiming to further dissect the content of CAF-CM to better understand the modes of intercellular communication that exist in this system. It is well established that virtually all cell types secrete sEVs into the extracellular environment (60), and sEVs secreted by CAFs have been demonstrated to modulate the survival of recipient cancer cells (17, 61). Thus, we focused on dissecting the functional characteristics of sEVs contained within NetG1^+^ CAF-CM for the ability to support the survival of PDAC cells *in vitro* during nutrient deprivation.

Taking this approach, we determined that compared with sEVs from NLFs, CAF-sEVs are capable of providing prosurvival benefits to nutrient-deprived PDAC cells. We also learned that NetG1 plays a necessary role in this system, as NetG1-deficient CAFs produced sEVs that phenocopied the inability of NLF-sEVs to support PDAC survival, while reexpression of NetG1 in NetG1^KD^ CAFs returned their tumor-supportive function. Furthermore, this NetG1-dependent survival benefit conferred by CAF-sEVs was accompanied by lower levels of PARP cleavage occurring in PDAC cells as well as increased Akt phosphorylation, a key event in prosurvival signal transduction pathways (46, 47). This suggested that NetG1^+^ CAF-sEVs are capable of stimulating survival pathways that protect recipient PDAC cells from undergoing nutrient-deprived induced apoptosis. In addition, we speculate that these tumor-supportive properties observed in CAF-sEVs could be partially attributed to the NetG1-dependent metabolite cargo provided to PDAC cells, of which Gln, a key amino acid used for survival in PDAC (10) that was shown to be dependent on NetG1 expression in CAF/ECM units (27), is significantly enriched compared with sEVs from NetG1^KD^ CAFs.

It was also the goal of this investigation to characterize populations of CAF-EVs based on known CAF markers, NetG1 (27) and Int.α_5_ (28), for the purpose of identifying a pathogenic profile from pancreatic stroma-derived EVs. sEV fractions are known to be enriched in exosomes and similarly sized vesicle structures, however this fraction is inherently heterogeneous (62). Through transmitted electron microscopy combined with double immunolabeling, we made the novel discovery of NetG1 in exomere-like (23) structures that are much smaller than canonical exosomes. Notably, NetG1 failed to colocalize with exosome marker CD81 (50). In contrast, Int.α_5_ did colocalize to CD81^+^ exosome-like structures, confirming our hypothesis that Int.α_5_’s subcellular localization to the multivesicular body in CAFs (28) would result in its packaging and secretion in exosomes. Similarly, NetG1’s cellular localization to the PM in CAF/ECM units and its secretion in subexosomal structures is in agreement with concurrent discoveries in the field which describe exomeres as having a separate biogenesis pathway from exosomes and as a consequence, possessing unique biochemical properties (23, 34). While the exact mechanism of biogenesis and secretion of exomeres remains under investigation, previous studies have suggested that these vesicles can “shed off” from the PM (23), in a way that distinguishes these from the canonical pathway by which exosomes are generated (e.g., involving endosomal trafficking to multivesicular bodies (63)). Furthermore, we generated an exomere-enriched DNP fraction and compared it with the exosome-enriched sEV fraction and noted that NetG1^+^ EVs are indeed highly represented in the DNP fraction. Previous studies have shown that DNP fractions are enriched in glycolytic components (22, 34), which served as a basis for using hexokinase1 and GAPDH as positive markers for our DNP fraction and suggest a potential metabolic role for the newly identified NetG1^+^ vesicles. In addition, exomere-like DNPs have been shown to be enriched with GPI-anchored proteins (23). These studies could explain our observation that NetG1, also being a GPI-anchored protein (26), was detected in tumor-promoting CAFs at the PM, and was sorted to DNPs.

Results from both the proteomic and metabolomic analyses highlighted the heterogeneity of the CAF-EV subtypes isolated. While it was unsurprising that both the sEV and DNP fractions included ECM-relevant components as supported by published work (64), it was interesting to note that unique ECM proteins were segregated to each of the EV fractions. For example, collagen alpha-2 was highly represented in DNPs while collagen alpha-1 and alpha-3 were enriched in sEVs. This observation is noteworthy as ratios of collagen chains in the ECM have been shown to inform pathologic states in cancers (65). Thus, further investigation into these trends may highlight specific EVs associated with stromal states. In addition, CAF-DNP fractions were enriched in proteins involved in cellular metabolism, which may serve to positively identify these types of EVs. In addition, these data may also provide some explanation as to how these DNPs exert a PDAC cell survival benefit. It has been previously reported that enzymes transferred via DNPs can continue to be active in recipient cells (22). For example, DNPs included transketolase, a thiamine pyrophosphate-dependent enzyme involved in the pentose-phosphate metabolic pathway (66) and an integral component of the glucose-repurposing pathway for generating NADPH, nucleic acid, and amino acids (67). LDHA, another key enzyme in glucose metabolism, was also highly represented in CAF-generated DNPs. LDHA has been reported to be overexpressed in cancer cells, and contributes to several oncogenic behaviors, the most relevant of which is maintaining cell survival under nutrient stress (68). This rationale suggests the possibility that the tumor-supportive function observed in the CAF-DNP fraction could, in part, be due to the delivery of functional glycolytic enzymes to PDAC cells, which will be the subject of future investigations into the functional significance of DNP cargo.

While both sEVs and DNPs performed a function in supporting the survival of nutrient deprived PDAC cells, the mechanisms that drive each are yet unknown and may differ. Metabolomic data demonstrated that CAF-sEVs have a NetG1-dependent metabolite profile which could play a role in their observed prosurvival behavior. Notably, sEVs have a significantly higher metabolite content compared with DNPs, suggesting that the prosurvival function observed in DNPs could be due to their protein content instead. Importantly, previous studies have demonstrated the ability of EVs to transfer functional enzymes to recipient cells (19, 34, 69, 70). Thus, it is possible that the observed DNP-mediated function could be attributed to the transfer of metabolism- and other stress response–related enzymes; while sEV-mediated survival could stem from metabolite content. In an *in vivo* context, the EV-mediated transfer of these types of metabolism-stimulating proteins and key metabolites, such as gln, from CAFs to PDAC could be a mechanism by which CAFs are able to provide prosurvival advantages during early disease development. Encouragingly, data collected in this study regarding the specific metabolites packed as NetG1^+^ CAF-generated sEV cargo matched our previous results reporting that CAFs modulate protumoral metabolism via two key proteins (27). The first was glutamine synthetase, known for the synthesis of gln and the second was VGLUT1, which is a well-characterized glutamatergic transporter responsible for loading presynaptic vesicles. Both proteins were shown to be modulated by and act downstream to NetG1 in PDAC CAFs and both were reported to be necessary for NetG1 protumoral function (27). Ultimately, it will be the goal of future studies to further elucidate the NetG1-modulated mechanisms responsible for sorting cargo and generating the novel EV subpopulations produced by NetG1^+^ CAFs.

We also note that losing NGL-1 in PDAC cells eliminated the benefit provided by CAF-EVs. Our previous work showed that NGL-1-deficiency in these cells causes a decrease in the amount of macropinocytosis mediated uptake of extracellular material, which can be one potential explanation for the loss of function in survival following CAF-sEV treatment. Interestingly, this function was reported to be independent of NetG1 (27). Then again, a NetG1/NGL-1–dependent material transfer was also reported (27), which was also evident in this study, based on results obtained using increased amounts of rNGL-1. These results could be due to the rNGL-1 engaging NetG1 on the EVs, and thus preventing an interaction with NGL-1 on the PDAC cells, and blocking subsequent uptake of EVs. Considered as a whole, there is reported evidence for a NetG1-independent mechanism of CAF-sEV–mediated survival regulated by macropinocytosis, as well as a NetG1-NGL-1–dependent mechanism further supported by results from this study.

Another exciting aspect of this study was finding that our *in vitro* data were representative of clinical subjects. We report evidence to suggest that the CAF markers identified in sEVs isolated from the CM of CAF/ECM units, cultured *in vitro,* were also detected in plasma from patients with PDAC. Moreover, we noted that levels of NetG1 and Int.α_5_ in sEVs are significantly higher in PDAC patient plasma compared with healthy age/sex-matched donors. This is important when considering that PDAC is a difficult disease to detect systemically. The development of a blood-based PDAC-associated EV profile would serve a much needed role in the early detection of this disease (71). Hence, our findings establish a basis for future in-depth studies that would aim to assess the potential of unique EV populations to act as indicators of pathologic stromal states. To this end, numerous recent studies have demonstrated that pancreatic stromal signatures could be indicative of PDAC and other cancers’ patient outcomes (27, 72–80).

EV research is a broad yet rapidly expanding field that continues to elucidate the diverse nature of EVs both from a functional, as well as a characteristic standpoint. In this study, we report the novel role of NetG1 in CAF-EV–mediated survival of nutrient-deprived PDAC cells, as well as early evidence for their potential as clinical biomarkers.

## Supplementary Material

Supplementary Figures S1-S9 Supplementary Figure 1. Confirmation that CAFs produce ECMs distinct from NLFs. Supplementary Figure 2. Direct co-culture of CAFs support PDAC cells during nutrient-deprivation. Supplementary Figure 3. Additional CAF cell lines generate sEVs that rescue PDAC cells from nutrient deprivation-induced apoptosis. Supplementary Figure 4. NetG1+ CAF-sEVs support PDAC cell survival in a NGL-1 dependent manner. Supplementary Figure 5. NetG1 expression in CAFs is necessary for sEV-mediated survival of nutrient-deprived PDAC cells. Supplementary Figure 6. NetG1 ablation does not affect the uptake of sEV cargo in PDAC cells. Supplementary Figure 7. sEV supernatant contains DNPs enriched with sub-exosome sized EVs. Supplementary Figure 8. Enriched gene ontology clusters from proteomic and metabolomic analysis comparing sEV and DNP fractions. Supplementary Figure 9. EV cargo requires transfer in intact vesicles to provide tumor-supportive effect.

Supplementary File 1 (proteomics) Spreadsheet including the proteomic analyses corresponding to the experiments indicated by the naming of the assorted tabs. Specific analysis parameters for each experiment are provided within the respective main figure legends.

Supplementary File 2 (statistical analyses) Spread sheet including a single tab per Figure panel conveying each graph's statistical analyses. Note that tabs are named accordingly. The tabs include the comprehensive statistical analyses that were performed for each experiment in which statistical significance was given. Each tab contains the full statistical output for its labeled experiment. The statistical test performed for each experiment is identified within the corresponding tab. Statistical tests were carried out using the software, Prism, version 7.05.

Supplementary File 3 (metabolomics) Spreadsheet including the metabolomic analyses corresponding to the experiments indicated by the naming of the assorted tabs. Specific analysis parameters for each experiment are provided within the respective main figure legends.
