# Supplementary material for: NetrinG1+ Cancer-Associated Fibroblasts Generate Unique Extracellular Vesicles that Support the Survival of Pancreatic Cancer Cells Under Nutritional Stress
Source: Cancer Res Commun. 2022 Sep 19;2(9):1017–36. doi: 10.1158/2767-9764.CRC-21-0147 (PMC9608356; doi:10.1158/2767-9764.CRC-21-0147)
Supplement: Supplementary Figures S1-S9 — Supplementary Figure 1. Confirmation that CAFs produce ECMs distinct from NLFs. Supplementary Figure 2. Direct co-culture of CAFs support PDAC cells during nutrient-deprivation. Supplementary Figure 3. Additional CAF cell lines generate sEVs that rescue PDAC cells from nutrient deprivation-induced apoptosis. Supplementary Figure 4. NetG1+ CAF-sEVs support PDAC cell survival in a NGL-1 dependent manner. Supplementary Figure 5. NetG1 expression in CAFs is necessary for sEV-mediated survival of nutrient-deprived PDAC cells. Supplementary Figure 6. NetG1 ablation does not affect the uptake of sEV cargo in PDAC cells. Supplementary Figure 7. sEV supernatant contains DNPs enriched with sub-exosome sized EVs. Supplementary Figure 8. Enriched gene ontology clusters from proteomic and metabolomic analysis comparing sEV and DNP fractions. Supplementary Figure 9. EV cargo requires transfer in intact vesicles to provide tumor-supportive effect. [file crc-21-0147-s01.docx]

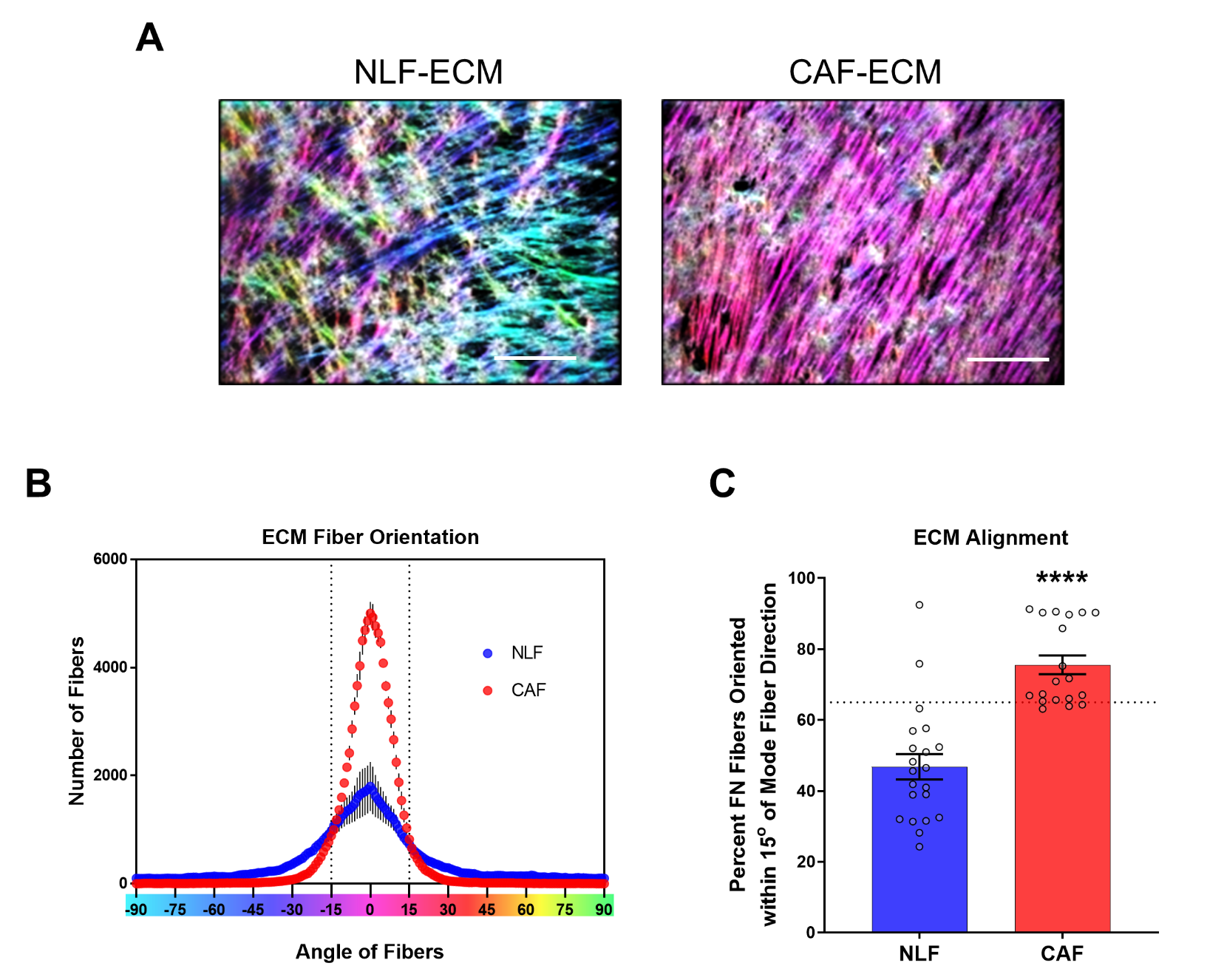


**Supplemental Figure 1:** *Confirmation that CAFs produce ECMs distinct from NLFs.* (A) Representative image of fibronectin fiber orientation measured via the orientation-J plugin in FIJI assigns a color gradient based on degree of orientation around a mode angle. Scale bars = 50 µm. (B) Histogram distribution of quantified ECM fiber orientation visualizing fiber alignment. The area denoted between the two dotted lines represents the number of fibers oriented at 15^º^ angles from the mode (normalized to 0^º^). (C) Percentage of fibers within 15^º^ of the mode (obtained from the area between dotted lines of panel (B) were compared to question significance in alignment. Red dotted line denotes 65% alignment that is indicative of myofibroblastic activation. n=3. Bars = standard error. Statistical test used: Unpaired t-test. A comprehensive list of statistical readouts is provided in the Supplemental File 2 (Tabs=Fig.S1C).


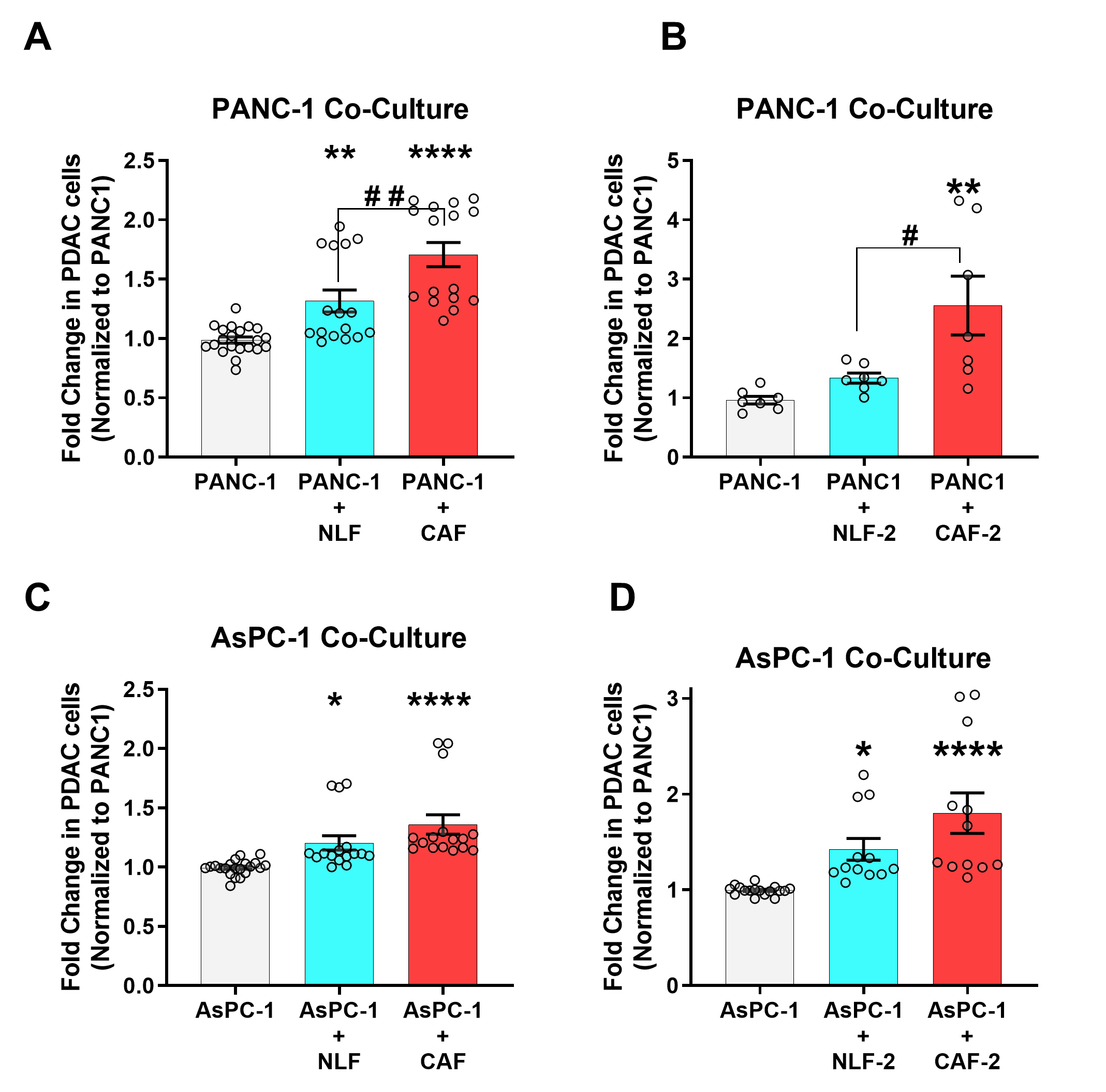


**Supplemental Figure 2:** *Direct co-culture of CAFs support PDAC cells during nutrient-deprivation.*

Direct co-culture of PDAC cells (PANC-1 A-B; AsPC-1 C-D) and CAF and NLF fibroblastic cells harvested from two independent patients. n=3. Bars = standard error. * denotes comparisons between experimental and PBS-treated condition (negative control), while # denotes comparisons between experimental conditions linked by connecting lines. Statistical test used: 1-way ANOVA, with multiple comparisons with Tukey’s correction. A comprehensive list of statistical readouts is provided in the Supplemental File 2 (Tabs=Fig.S2A, S2B, S2C, S2D).


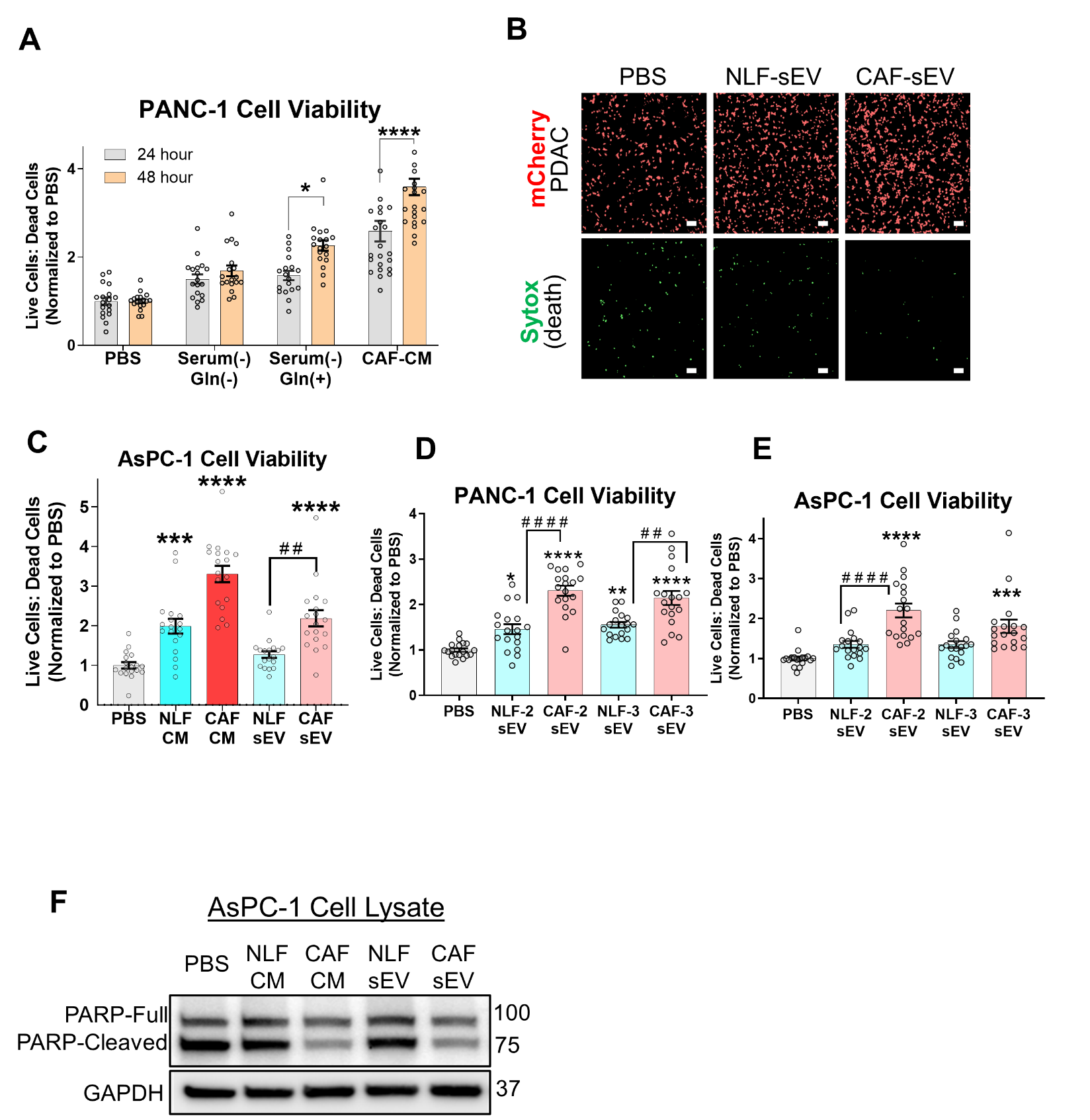


**Supplemental 3:** *Additional CAF cell lines generate sEVs that rescue PDAC cells from nutrient deprivation-induced apoptosis.* (A) Cell viability assay comparing 24- and 48-hour endpoints, post-treatment with PBS, starvation media, Glutamine-supplemented starvation media, or CAF-CM. n=3 biological replicates; each replicate consists of 6 technical repeats. All repeats per replicate were normalized to the corresponding PBS-treated condition. Bars = standard error. Statistics: 2-way ANOVA, using Sidak’s correction. * denotes comparison between conditions linked by connecting lines. Comprehensive statistics in Supplemental File 2 (Tab=Fig.S3A). (B) Representative fluorescent images of PANC-1 cells 48 hours post-treatment, as used to quantify cell viability. Live cells (shown in red) observed via cellular expression of mCherry; cell death (shown in green) observed via Sytox Blue membrane-impermeable nucleic acid stain (i.e. only cells with compromised membranes will be labeled). Scale bars = 100 µm. (C) Cell Viability assay as in Figure 2E using AsPC-1 cells at 48 hours post-treatment with CM or sEV from NLFs and CAFs. (D-E) PANC-1(D) and AsPC-1(E) viability assays from Figure 2E, using sEVs collected from 2 additional patient-derived matched fibroblast lines (i.e. NLF-2/CAF-2 derived from same patient, and NLF-3/CAF-3 from another patient). (F) Representative western blots of AsPC-1 cell lysates 48 hours post-treatment with PBS, CM, or sEVs from NLF or CAFs; probing for full-length and cleaved PARP. GAPDH used as a loading control.


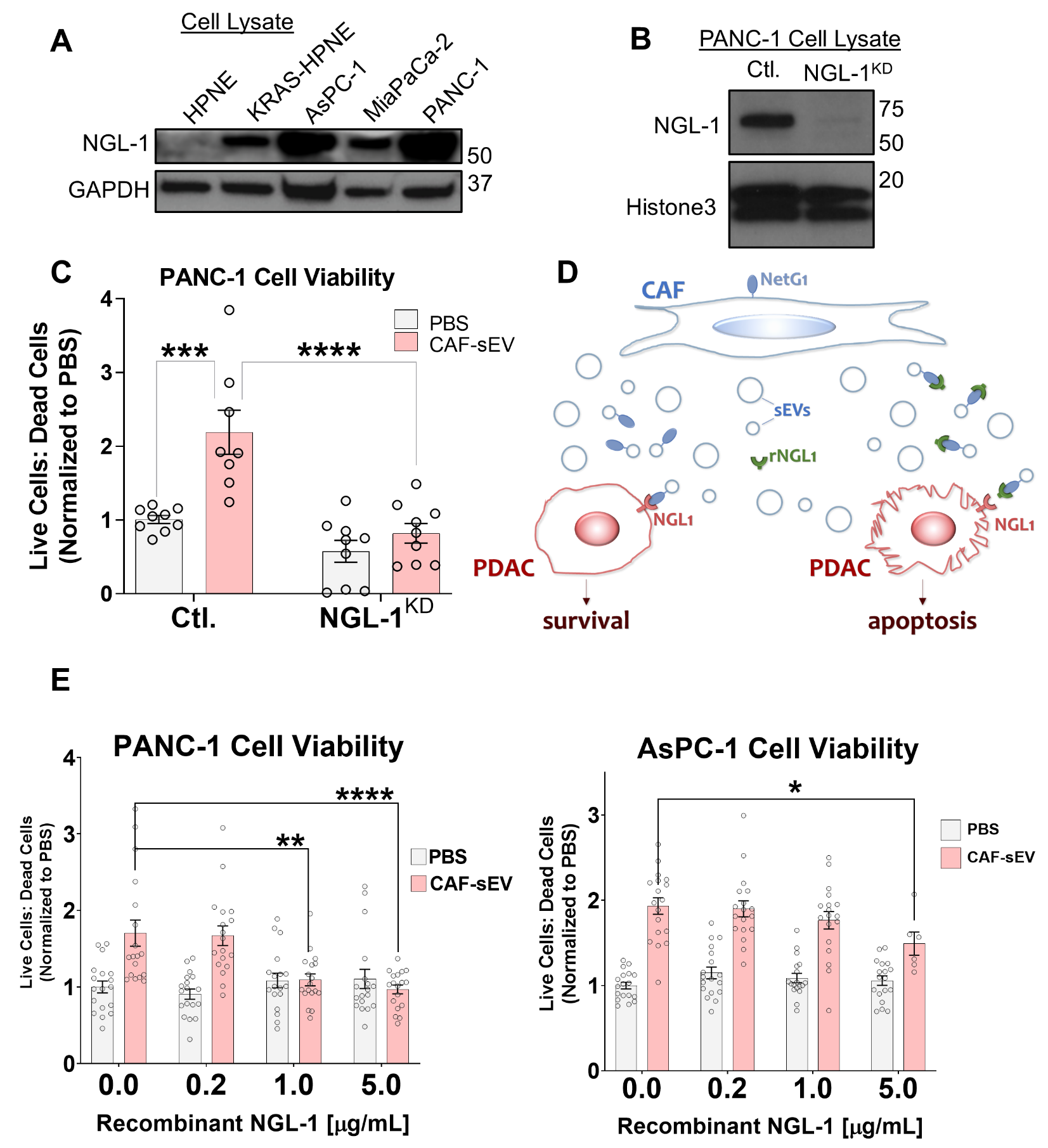


**Supplemental Figure 4:** *NetG1+ CAF-sEVs support PDAC cell survival in a NGL-1 dependent manner.* (A) Representative western blot depicting NGL-1 expression in assorted pancreatic epithelial cell lysates. GAPDH as protein loading control. (B) Representative western blot of control vs NGL-1^KD^ PANC-1 cell lysates. Histone 3 as protein loading control. (C) Cell viability of Ctl. versus NGL-1^KD^ PANC-1 cells 48-hours post-treatment with CAF-sEVs. n=3 biological replicates; each replicate consists of a minimum of 6 technical repeats. All repeats per replicate were normalized to the mean of the corresponding Ctl. PANC-1 PBS-treated condition. (D) Schematic representing the experimental design in (E); sEVs from NetG1^+^ CAFs were isolated, incubated with recombinant NGL-1 for 20 minutes at room temperature and administered to nutrient-deprived NGL-1+ PDAC cells. (E) Dose responsive viability assay of PDAC cells treated with CAF-sEVs incubated with recombinant NGL-1. For all graphs: n=3 biological replicates; each biological replicate included 3 technical repeats. Technical repeats for each biological replicate were normalized to the 0.0 µg/mL PBS-treated condition. For C and E: Bars = Standard Error. Statistics = 2-way ANOVA, with multiple comparisons using Tukey’s correction. * Comparing between conditions noted by connecting lines. Comprehensive statistical readouts provided in Supplemental File 2 (Tabs = Fig.S4C, S4E).


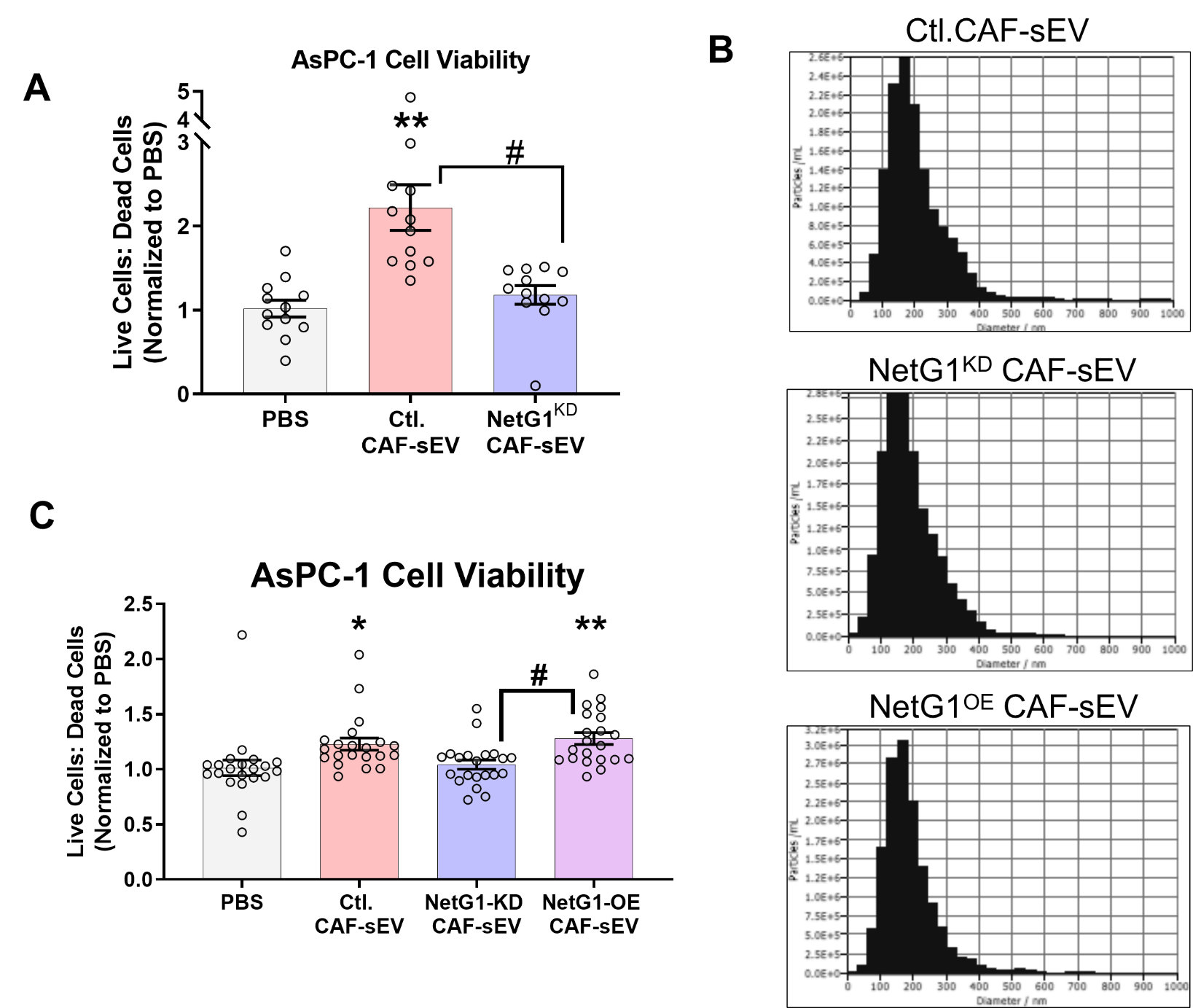


**Supplemental Figure 5:** *NetG1 expression in CAFs is necessary for sEV-mediated survival of nutrient-deprived PDAC cells.* (A) Cell viability assay as in Figure 3D using AsPC-1 cells at 48 hours post-treatment with sEVs from Ctl. or NetG1^KD^ CAFs. n=3 biological replicates. (B) Representative NTA histograms of sEVs from Ctl., NetG1^KD^, and NetG1^OE^ CAFs ; using the ZetaView platform. (C) Cell viability assay as in Figure 3I using AsPC-1 cells at 48 hours post-treatment with sEVs from Ctl., NetG1^KD^ , or NetG1^OE^ CAFs. n=3 biological replicates. For B and C: each replicate consists of 6 technical repeats. All repeats per replicate were normalized to the mean of the corresponding PBS-treated condition. Bars = standard error. Statistics: 1-way ANOVA, with multiple comparisons using Tukey’s correction. * Compared to PBS (negative control), # comparing between conditions noted by connecting lines. Comprehensive statistical readouts provided in Supplemental File 2 (Tabs=Fig.S5A, S5C).


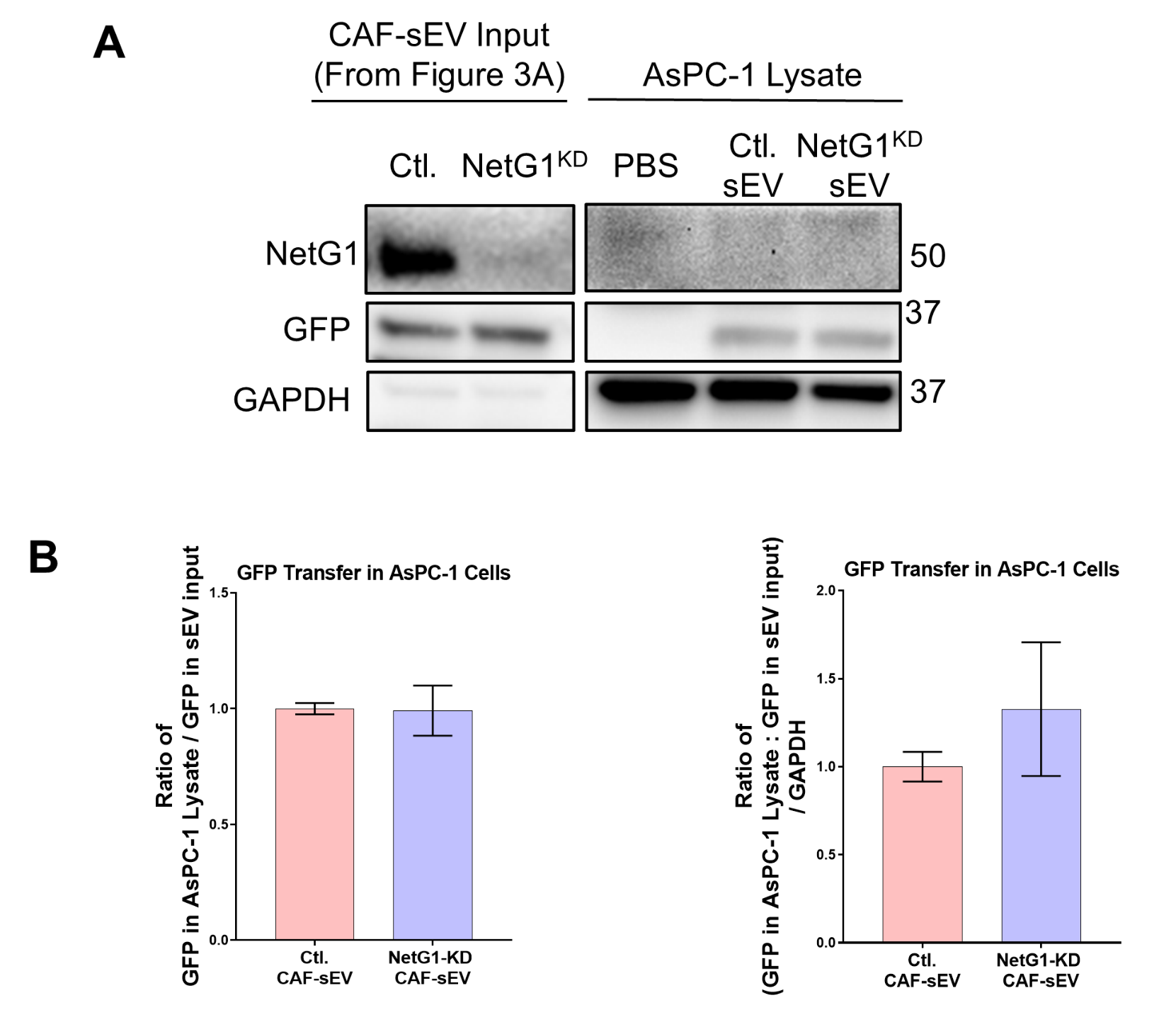


**Supplemental Figure 6:** *NetG1 ablation does not affect the uptake of sEV cargo in PDAC cells.* (A) Representative western blots showing input levels of GFP in sEVs (Left panel) and amount of GFP in AsPC-1 cell lysates 24 hours post treatment (Right panel). GAPDH as protein loading control in cell lysates. (B) GFP transfer quantified from the optical density of digitized western blots in (A) using the software ImageJ. (Left) Ratio of GFP in PDAC cell lysates to the GFP in the sEV input, and (Right) Values in left graph normalized to the GAPDH of the respective cell lysate. Results normalized to the Ctl. sEV treated condition; n=2. Bars=standard deviation.


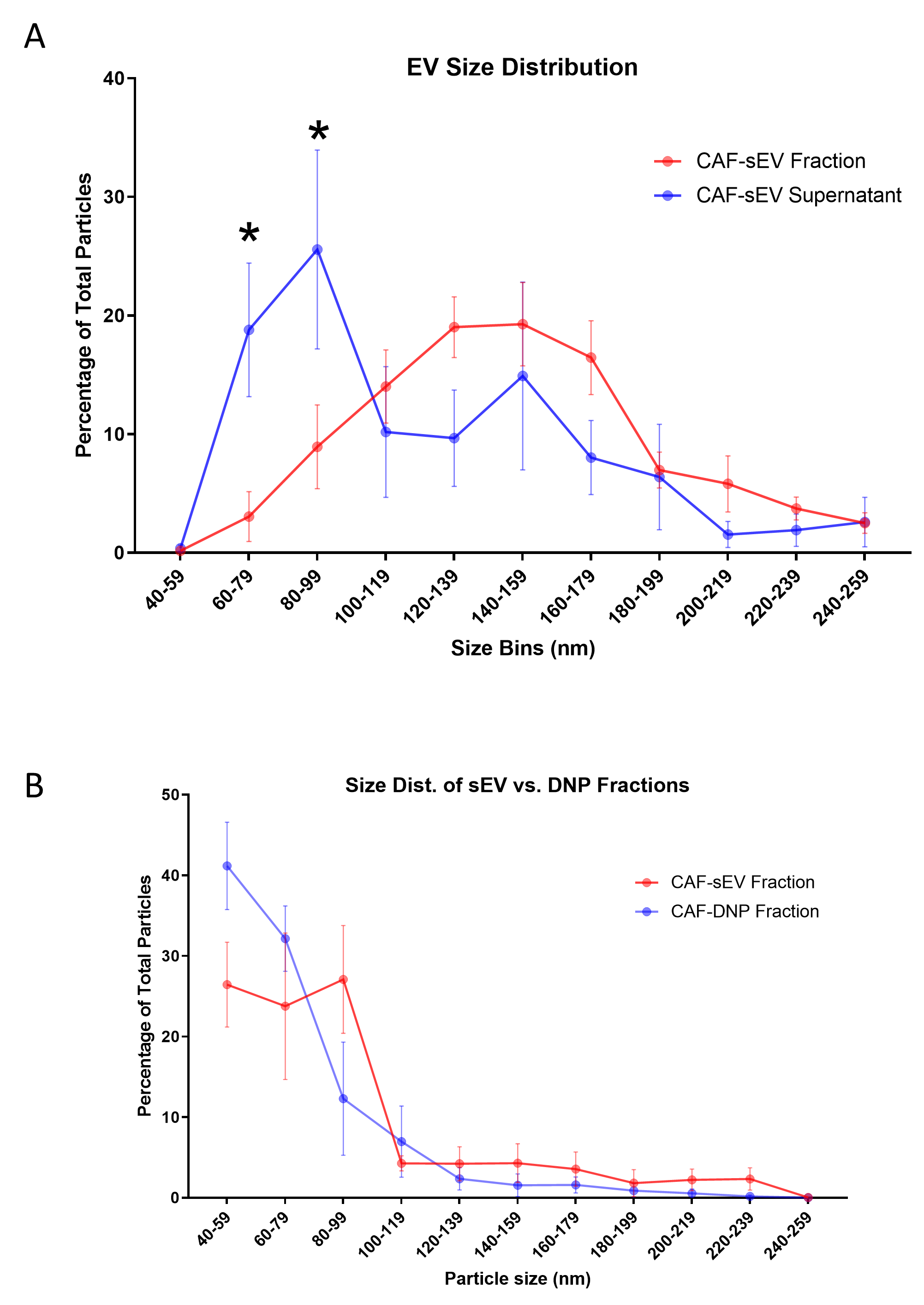


**Supplemental Figure 7:** *sEV supernatant contains DNPs enriched with sub-exosome sized EVs.* (A-B) NTA distribution of particles isolated from equal volumes of (A) CAF-sEV and sEV supernatant and (B) CAF-sEV and CAF-DNP fractions. Reported counts of particles sizes were grouped by 20 nm range bins (from 40 nm- 250 nm), and the percentage of each bin compared to the total EV count was graphed. Performed on the NanoSight platform, a cutoff of 40 nm was arbitrarily used based on the limitations of the NTA hardware’s detection threshold. n=3 biological replicates. Statistics: 2-way ANOVA with multiple corrections using Sidak’s correction. * denotes significance between conditions at the indicated bin size. Comprehensive statistical readouts provided in the Supplemental File 2 (Tabs=Fig.S7A, S7B).


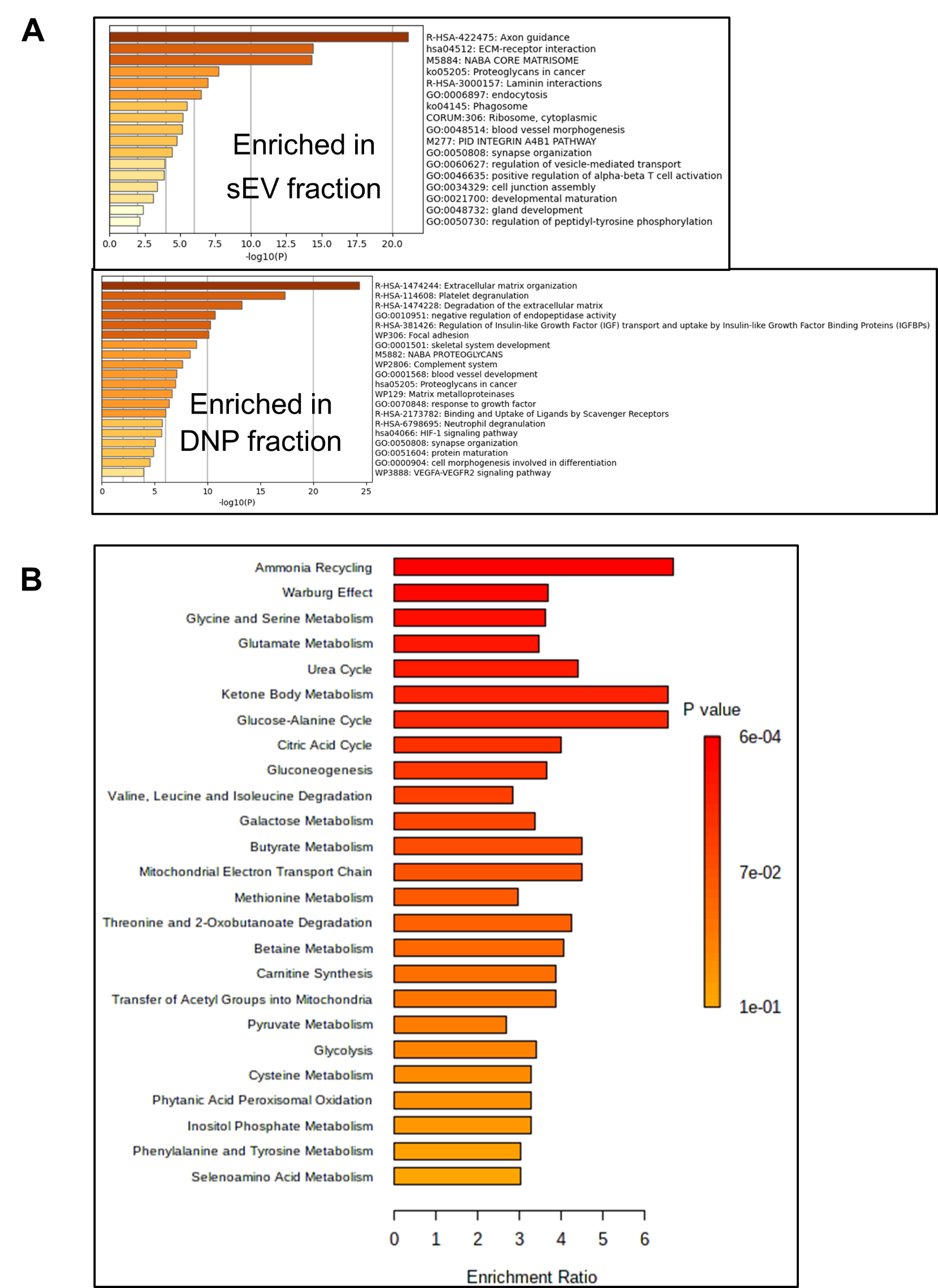


**Supplemental Figure 8:** *Enriched gene ontology clusters from proteomic and metabolomic analysis comparing sEV and DNP fractions.* (A) Following LFQ experimental analysis, all proteins whose p-values indicated significantly enriched levels in sEVs (top) or DNP (bottom) were identified for enriched terms including GO/KEGG terms, canonical pathways, and hallmark gene sets. Ontology clusters were sorted, calculated, and ranked by p-values using the Metascape.org gene annotation and analysis resource. Full readout of pathway enrichment analysis can be found in Supplemental File 1 (Tabs=Fig.S8A sEV-up Pathways, Fig.S8A DNP-up Pathways). (B) Pathway enrichment using metabolomics data in Figure 6F comparing sEVs to DNPs was queried using the MetaboAnalyst and the top 25 significant pathways enriched in sEVs are shown.

**Supplemental Figure 9:** *EV cargo requires transfer in intact vesicles to provide tumor-supportive effect.* Cell viability assay of PANC-1 cells at 48 hours post-treatment with the indicated fractions of CAF-secreted material. Fractions were collected and either remained at room temperature (Intact) or incubated at 100^o^C for 15 min and passed through a 3kD pore-sized centrifugal filter to remove denatured protein aggregates (Boiled). n=3 biological replicates. Each biological replicate consists of 6 technical repeats. All repeats per replicate were normalized to the mean of the corresponding PBS-treated condition (negative control). Bars=standard error. Statistics: 2-way ANOVA, with multiple comparisons using Sidaks’s correction. * Comparing between conditions noted by connecting lines. Comprehensive statistical readouts provided in the Supplemental File 2 (Tab=Fig.S9).

**Legends for Supplementary Data Files:**

Supplemental File 1 (proteomics): Spreadsheet including the proteomic analyses corresponding to the experiments indicated by the naming of the assorted tabs. Specific analysis parameters for each experiment are provided within the respective main figure legends.

Supplemental File 2 (statistical analyses): Spread sheet including a single tab per Figure panel conveying each graph's statistical analyses. Note that tabs are named accordingly. The tabs include the comprehensive statistical analyses that were performed for each experiment in which statistical significance was given. Each tab contains the full statistical output for its labeled experiment. The statistical test performed for each experiment is identified within the corresponding tab. Statistical tests were carried out using the software, *Prism,* version 7.05.

Supplemental File 3 (metabolomics): Spreadsheet including the metabolomic analyses corresponding to the experiments indicated by the naming of the assorted tabs. Specific analysis parameters for each experiment are provided within the respective main figure legends.
